# Lateral gain is impaired in macular degeneration and can be targeted to restore vision in mice

**DOI:** 10.1038/s41467-022-29666-x

**Published:** 2022-04-20

**Authors:** M. Rizzi, K. Powell, M. R. Robinson, T. Matsuki, J. Hoke, R. N. Maswood, A. Georgiadis, M. Georgiou, P. R. Jones, C. Ripamonti, F. M. Nadal-Nicolás, M. Michaelides, G. S. Rubin, A. J. Smith, R. R. Ali

**Affiliations:** 1 UCL Inst. of Ophthalmology, London, UK; 2 Centre for Cell and Gene Therapy, King’s College London, London, UK; 3 CRS Ltd, Rochester, UK; 4 Departamento de Oftalmología, Facultad de Medicina, Universidad de Murcia and IMIB-Arrixaca, Murcia, Spain; 5 Retinal Neurophysiology Section, National Eye Institute, NIH, Bethesda, MD USA; 6 NIHR Biomedical Research Centre at Moorfields Eye Hospital and UCL Institute of Ophthalmology, London, UK

**Keywords:** Macular degeneration, Experimental models of disease, Cellular neuroscience

## Abstract

Macular degeneration is a leading cause of blindness. Treatments to rescue vision are currently limited. Here, we study how loss of central vision affects lateral feedback to spared areas of the human retina. We identify a cone-driven gain control mechanism that reduces visual function beyond the atrophic area in macular degeneration. This finding provides an insight into the negative effects of geographic atrophy on vision. Therefore, we develop a strategy to restore this feedback mechanism, through activation of laterally projecting cells. This results in improved vision in Cnga3^−/−^ mice, which lack cone function, as well as a mouse model of geographic atrophy. Our work shows that a loss of lateral gain control contributes to the vision deficit in macular degeneration. Furthermore, in mouse models we show that lateral feedback can be harnessed to improve vision following retinal degeneration.

## Abstract (web summary)

Treatments to rescue vision are currently limited. Here, the authors identify a cone-driven gain control mechanism that reduces visual function beyond the atrophic area in humans. They also show that activating laterally projecting cells results improved vision in two mouse models of retinal degeneration.

## Introduction

Input from laterally-projecting cells is a prominent feature of the visual system^1^, with many identified cell types and specific mechanisms providing modulatory feedback to laterally displaced cells and circuits^2,3^. Lateral input is present in many circuits of the retina, including at the first synapse of the visual system^4–11^, where horizontal cells modulate photoreceptor output over large distances, aided by long axonal plexuses and networks of gap junction-connected cells^4,12–15^. Notably, lateral interactions have also been observed at the perceptual level and have been shown to have a substantial impact on visual function^16,17^. However, less has been done to investigate how the loss of lateral feedback, caused by focal photoreceptor degeneration, impacts remaining vision. Given the importance of lateral input in normal visual function, it has been hypothesized that its loss would affect the function of surviving retinal cells in adjacent regions^18–20^. An anatomically defined scotoma would therefore contribute to a wider functional scotoma. For example, macular degeneration is a condition where a chronic, progressive degeneration occurs in the central part of the retina (macula). In one of its most common end-stage forms, it leads to a scotoma caused by photoreceptor loss (geographic atrophy). Therapeutic strategies that could restore lateral feedback would therefore reduce this functional scotoma and potentially improve vision in conditions such as macular degeneration^21^. The loss of lateral feedback may also exacerbate the progression of degenerative conditions, as suggested by data from transgenic mice, which lack lateral inputs in the outer retina^12^. Together, these issues highlight the importance of studying lateral feedback in degeneration. In this study, we aimed to quantify both the nature and magnitude of lateral input on normal visual perception and its contribution to the visual deficit in patients with macular degeneration. We also investigated the circuitry mediating this effect in a range of patients lacking function in specific photoreceptor subtypes. These patients led us to identify a retinal cell type, H1 horizontal cell^4,12,22,23^, as an important mediator of the effects of a scotoma on the surviving retina. This data allowed us to test in mice a therapeutic strategy to restore H1 lateral feedback and show improved vision following focal photoreceptor loss.

## Results

### Lateral gain control optimizes visual perception to contextual luminance in subjects with normal vision

**Fig. 1:**
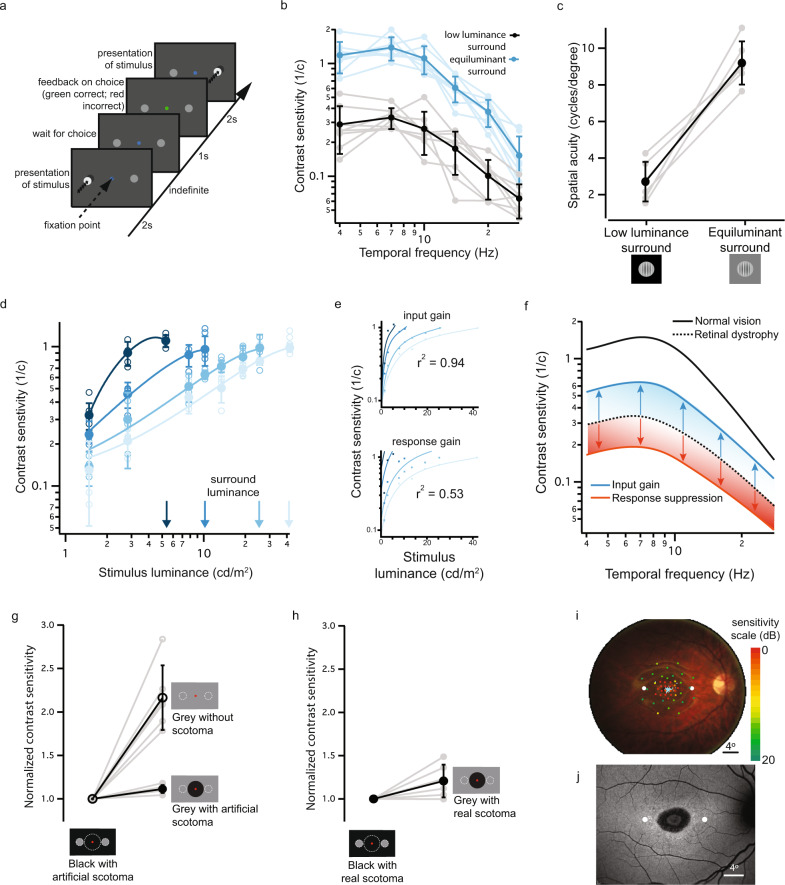
A lateral adaptive input improves contrast encoding in human subjects. **a** Schematic of the experimental procedure. A two-alternative forced-choice task was used to measure temporal and spatial contrast sensitivity thresholds. **b**, **c** Vision was improved by an equiluminant (27.7 cd/m^2^) vs low luminance (0.15 cd/m^2^) surround in normal vision subjects. Temporal contrast sensitivity shown in **b** (*n* = 8 normal vision subjects; 4 Hz *p* = 0.0018; 7 Hz *p* = 0.0010; 10 Hz *p* = 0.0046; 14 Hz *p* = 0.0043; 20 Hz *p* = 0.0009; 28 Hz *p* = 0.055; one-way ANOVA, with Tukey post hoc multiple comparison test) and acuity in panel **c** (*n* = 6 normal vision subjects; *p* = 0.0004; two-sided paired *t*-test). **d** Surround luminance values (blue arrows) shift contrast encoding functions (corresponding blue color) along the x-axis, but maximum contrast sensitivity remains comparable (*n* = 4 subjects). **e** An input gain computation (see Methods) captures the shift between luminance conditions better (top graph, *r*^2^ = 0.94) than a response gain computation (bottom graph, *r*^2^ = 0.53). **f** Schematic demonstrating the effect of two types of lateral inhibition on reduced contrast sensitivity (dashed line). Response suppression would further reduce contrast sensitivity (red); input gain can enhance contrast sensitivity (blue). **g** An artificial scotoma (see Methods) significantly worsened contrast sensitivity thresholds measured with equiluminant surrounds (*n* = 6 normal vision subjects, data normalized to values with low luminance surround). **h** Measurements in patients with Stargardt disease reveal a similar effect to an artificial scotoma (cfr. Fig. 1g, *n* = 6 patients; data normalized to values with low luminance surround). **i** Functional mapping of the degenerating area in patients with Stargardt disease by microperimetry. Color-coded scores (in dB) are superimposed on a fundus reflectance image. Cyan pixels indicate fixation measurements. Sensitivity scores guided the placement of stimuli at the edge of the functional scotoma (indicated by circular markers). **j** Fundus autofluorescence imaging (486 nm) of the retina presented in panel **h**, showing the extent of the macular lesion. Filled circles indicate the contrast sensitivity measurement locations. All contrast sensitivity values are calculated as 1/contrast threshold (1/c). Error bars denote standard deviations around mean values. See Supplementary Table 1 for all calculated *p* values. Source data are provided as a Source Data file.

The diverse functions of the nervous system arise from circuits with unique response properties and spatial organization. These responses can be shaped by opponent mechanisms, which can either scale the response (response suppression^4,24^) or scale the input (input gain control^5^), (Supplementary Fig. 1). Both mechanisms have been alternatively hypothesized for laterally-projecting neurons in the visual system^16,17,23,25,26^, including at the level of photoreceptor synapses^4–6,8–11,24^. Response suppression has the effect of reducing the maximum response to light in target cells, whereas gain control scales the response to keep it proportional with environmental luminance. We measured the effect of surround input on individuals with normal vision, using a two-alternative forced-choice visual task (Fig. 1a) and tested which of the two models, input gain vs response suppression, best describes the data. Both contrast sensitivity and spatial acuity were affected by surround luminance (Fig. 1b, c and Supplementary Fig. 2) and both lower and higher luminance surround similarly affected vision (Supplementary Figs. 3–5), consistent with previous work^26^. We found that a gain control model best fit the data (Fig. 1d, e), suggesting that surround input optimizes vision to contextual luminance. Given these results, photoreceptor degeneration may deprive spared adjacent areas of a gain control mechanism. This would have evident therapeutic relevance, as it indicates that reinstatement of lateral input would not further suppress but may instead improve remaining visual function, by optimizing responses for contextual luminance (Fig. 1f). Laterally-projecting circuits may therefore be a potential target to improve vision following photoreceptor degeneration.

### A reduction of lateral feedback negatively impacts remaining vision in individuals with normal vision and with macular degeneration

Since photoreceptor loss is local in disorders such as macular degeneration, we probed what would happen if only part of the lateral inputs were missing. To make a valid comparison, we kept stimulus location and mean luminance constant and tested contrast sensitivity with and without an adjacent artificial scotoma. While an equiluminant surrounded improved contrast sensitivity in subjects with normal vision in absence of the scotoma, this improvement was drastically impaired by the presence of the scotoma (Fig. 1g and Supplementary Fig. 6), consistent with previous work^27^. This result indicates that localized loss of lateral input is sufficient to affect remaining vision in nearby regions of the visual field. We then assessed a group of subjects with Stargardt disease^28^, where the scotoma is instead caused by photoreceptor degeneration (Fig. 1i, j and Supplementary Fig. 7) and again found a reduction in lateral gain comparable to the effect of the artificial scotoma in healthy subjects (Fig. 1h and Suppl. Figs. 8 and 9). Together, these results show that a scotoma affects visual processing beyond its border, depriving surrounding areas of lateral gain control input and worsening the quality of remaining vision as a result. Importantly, the effect is substantial (Fig. 1c, d, g), equivalent, in terms of visual acuity, to five lines on a logMAR standard visual acuity chart.

### Specific photoreceptor mutations in human subjects indicate rods as an important target of lateral gain modulation

**Fig. 2:**
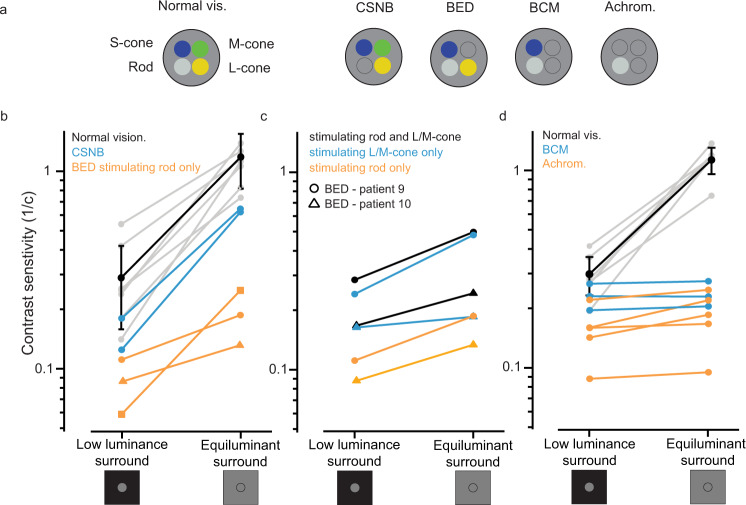
Specific photoreceptor mutations in patients identify the cell types involved in lateral adaptive feedback. **a** Schematic showing the contributing photoreceptor types in normal vision subjects, congenital stationary night blindness (CSNB, cone-only vision), Bornholm eye disease (BED, lack of one cone opsin; M-opsin for two of three patients tested, L-opsin for one patient) blue cone monochromatism (BCM, rod, and S-cone vision), achromatopsia (rod-only vision). **b** Selective stimulation of rod photoreceptors in two BED patients (*n* = 2 patients; orange traces) or cone photoreceptors in two CSNB patients (*n* = 2 patients; blue traces) showed a positive rather than suppressive effect. Comparative data for normal vision subjects is shown in black (*n* = 8 subjects, data also shown in Fig. 1b). **c** An equiluminant surround improved vision in two Bornholm eye disease patients using rod isolating (orange traces), cone isolating (blue traces), and rod-cone stimuli (black traces). **d** An equiluminant surround provided only a marginal improvement over a mismatched surround in patients with achromatopsia (*n* = 5 patients, orange traces) and blue cone monochromatism (*n* = 3 patients, blue traces), indicating an essential role for L/M cone photoreceptors in driving the effect. All contrast sensitivity values are calculated as 1/contrast threshold (1/c). Error bars denote standard deviations around mean values. Source data are provided as a Source Data file.

We then aimed to identify a cell type that might contribute to this lateral gain control and that, following photoreceptor degeneration, might be targeted to reverse the negative effect of a scotoma on vision. We tested vision in subjects with specific photoreceptor deficits (Fig. 2a) to characterize the role of photoreceptors in these interactions. In patients with cone-only vision (congenital stationary night blindness, CSNB), we found that engaging lateral feedback with an equiluminant surround improved contrast sensitivity, suggesting the importance of lateral feedback in optimizing cone-mediated vision (Fig. 2b, blue lines). In patients with only three functioning opsins (Bornholm eye disease, BED), silent substitution^29^ allowed selective stimulation of rod or cone photoreceptors within the stimulus area. In these subjects, we made the interesting observation that engagement of lateral input improved not only cone-mediated vision but also rod-mediated vision (Fig. 2b, c cone stimulation: blue lines (panel c), rod stimulation: orange lines (panels b and c); Supplementary Fig. 10). It has long been recognized that the saturation of the sensitive rod system in daylight affects the signaling in cone pathways upon which it impinges^4^. Our results indicate that this issue is not resolved by silencing rod photoreceptors but by providing them with lateral gain control input that improves their function.

The effect of lateral feedback was driven by cones but not rods in the surrounding area: we found an improvement in vision in patients where surround luminance engaged L/M cones (Fig. 2b, c and Supplementary Fig. 11), but not in patients where it could engage only rods (achromat patients) or rods and S-cones (blue cone monochromats) (Fig. 2d).

**Fig. 3:**
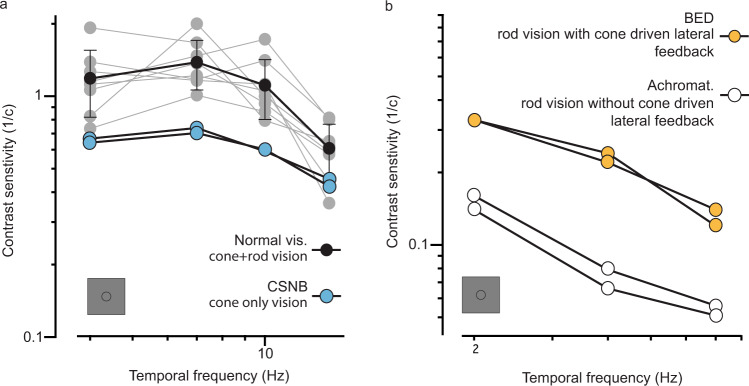
Rod photoreceptors contribute to daylight vision and receive adaptive feedback from cones. Contrast sensitivity functions were measured using temporally modulated stimuli in the presence of an equiluminant surround. **a** Contrast sensitivity functions in normal vision subjects, where vision is cone + rod-mediated (black markers, data also shown in Fig. 1b, *n* = 8 subjects) and in two patients (*n* = 2 patients) with congenital stationary night blindness (blue markers), with cone-only vision indicating that rod photoreceptors make a positive contribution to vision in daylight levels, almost doubling contrast sensitivity. **b** Contrast sensitivity functions for rod photoreceptors, obtained by silent substitution methods. In two tested BED patients (*n* = 2 patients, orange markers), where L/M cones can drive lateral feedback to rods, contrast sensitivity was better than in two tested achromatopsia patients (*n* = 2 patients; white markers), where rods receive no lateral feedback. All contrast sensitivity values are calculated as 1/contrast threshold (1/c). Error bars denote standard deviations around mean values. Source data are provided as a Source Data file.

Consistent with cones optimizing rod vision in daylight, we noticed that cone-only vision in CSNB patients^30^ was consistently worse (~2-fold) than rod+cone vision of normal vision subjects, indicating that rods make a positive contribution to daylight vision (Figs. 2b and 3a and Supplementary Fig. 11). We also observed, using a rod-only stimulus, that rod-mediated vision was better in BED patients, in which cones are functioning than in achromat patients, in which there is no cone-driven feedback (Fig. 3b). Taken together, these findings provide a coherent set of data, which indicates that in human vision L/M cones provide lateral gain control to both cones and rods.

### Gain of function experiments in horizontal cells demonstrate improved vision in Cnga3^−/−^ mice

**Fig. 4:**
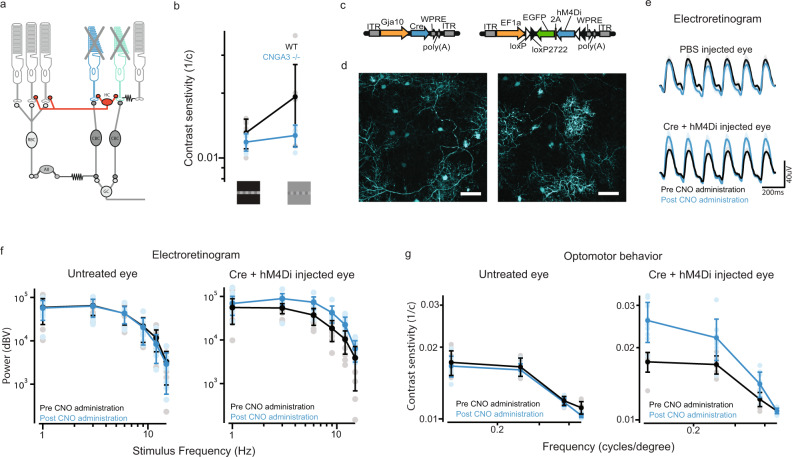
Restoration of lateral feedback to rod photoreceptors improves vision in a mouse model lacking cone function. **a** Schematic showing the loss of cone derived lateral feedback from H1 horizontal cells in a mouse model of achromatopsia. Horizontal cells would normally provide cone-driven feedback to laterally displaced rods, directly modulating their output onto cone pathways. **b** Contrast sensitivity measurements were obtained for wild-type (black trace, *n* = 8 mice) and Cnga3^−^^/−^ (*n* = 4 mice, blue trace) using a modified optomotor behavior paradigm driven by a 1° height sinusoidal stimulus with either an equiluminant or low luminance surround. An equiluminant surround increased contrast sensitivity in wild-type but not Cnga3^−/−^ mice, similar to human subjects (Fig. 2d). **c** Expression of hM4Di in horizontal cells was achieved by co-injection of a Gja10-Cre-expressing vector and a floxed hM4Di-GFP-expressing vector. **d** An example image of hM4Di-GFP expression in horizontal cells in mouse retina (wholemount, scale bar = 50 μm). **e** Chemogenetic activation of horizontal cells improves outer retina function in the Cnga3^−/−^ mouse model of achromatopsia. Averaged electroretinogram traces were recorded during the presentation of a 1 s stimulus with a 6 Hz sinusoid modulation. Tests were repeated before (black trace) and after (blue trace) intraperitoneal injection of the hM4Di activator CNO (mean average from *n* = 9 treated eyes and *n* = 9 untreated eyes from nine mice; shaded regions denote standard error). **f** Fourier analysis of electroretinogram responses in Cnga3^−/−^ mice to stimuli with different temporal frequencies at 0.1 cd/m^2^, before (black traces) and after (blue traces) CNO injection (*n* = 9 eyes from nine mice). Significant changes in contrast sensitivity were found after CNO administration in hM4Di treated eyes only (two-way ANOVA, with Sidak post hoc multiple comparison test 3 Hz, *p* = 0.0001; 6 Hz, *p* = 0.0002; 9 Hz, *p* = 0.0001; 12 Hz, *p* = 0.0004; 15 Hz, *p* = 0.0004). **g** Optomotor behavior measured improved contrast sensitivity at several spatial frequencies following administration of CNO in hM4Di treated eyes only (*n* = 9 eyes from nine mice; two-way ANOVA, with Sidak post hoc multiple comparison test; 0.128 cycles/degree, *p* < 0.0001; 0.25 cycles/degree, *p* = 0.0007; 0.381cycles/degree, *p* = 0.0004). See Supplementary Table 1 for all calculated *p* values. Error bars denote standard deviations around mean values. Source data are provided as a Source Data file.

**Fig. 5:**
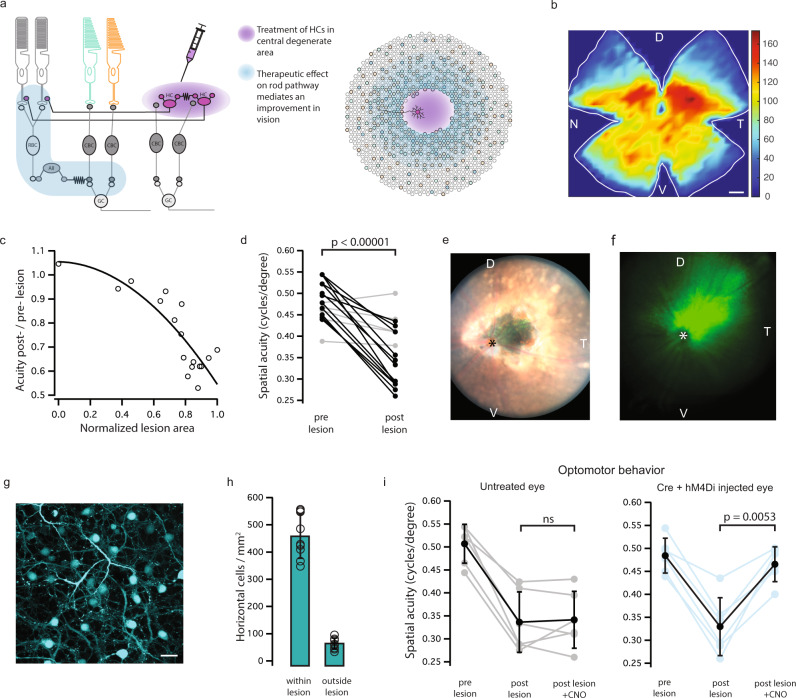
Improved vision following the restoration of lateral input to rod photoreceptors in a mouse model of focal photoreceptor loss. **a** Schematic showing the therapeutic strategy to improve remaining vision following the loss of lateral feedback. H1 horizontal cells provide feedback to laterally displaced rods, modulating their output onto cone pathways (left panel). Following focal photoreceptor degeneration, lateral input to surviving photoreceptors can be restored by transduction of horizontal cells with a hyperpolarizing actuator. **b** Ganglion cell density in WT mouse retina (Brn3a immunohistochemistry); color scale indicates RGC density/bin (bin: 0.1 mm^2^). D dorsal, V ventral, T temporal, N nasal. Scale bar: 800 μm. **c** Lesions in the dorso-ventral quadrant of the retina reduced the acuity threshold of WT mice. Plot shows acuity drop versus lesion size for individual eyes (fitted curve *r*^2^ = 0.73, *n* = 17 eyes). **d** Reduction in acuity following lesion for all eyes tested (*n* = 17 eyes; *p* < 0.00001; two-sided paired *t*-test,). Gray indicates lesioned area <30th percentile; (*n* = 5 eyes), black >30th percentile (*n* = 12 eyes). **e** Example fundoscopy image of WT mouse retina, showing a lesion in the dorsal-temporal quadrant and hM4Di-GFP-transduced cells. Asterisk indicates the location of the optic nerve, orientation indicated by D dorsal, V ventral, T temporal. **f** Injection of hM4Di construct targeted to the lesion. Average hM4Di-GFP-expression in retinal fundus images (*n* = 15 eyes) showing strong expression in the dorso-temporal quadrant. Asterisk indicates the location of the optic nerve, the orientation of the retinal image is indicated by the letters D dorsal, V ventral, T temporal. **g** Whole-mount confocal image of lesioned and transduced WT mouse retina showing dense labeling of horizontal cells in a region depleted of photoreceptors (scale bar; 20 μm). **h** Density of viral transduction was higher within the lesioned area than outside the lesion border (*n* = 9 eyes). **i** Following lesion of the dorsal-temporal retina, CNO administration significantly improved spatial acuity in h4MDi injected eyes only (right panel, *n* = 6 eyes from six mice; *p* = 0.0053, one-way repeated measures ANOVA with Tukey post hoc multiple comparison test). See Supplementary Table 1 for all calculated *p* values. Error bars denote standard deviations around mean values. Source data are provided as a Source Data file.

We then investigated what cell type could mediate the lateral effect we observed and could be targeted for therapeutic purposes. Diverse classes of laterally-projecting cells are known to mediate feedback within the retina. Horizontal cells have subtypes with specific cone-to-cone and cone-to-rod connectivity, with long-ranging axons and synaptic mechanisms consistent with input gain control feedback^13–15,31^. These features make them likely candidates contribute to the lateral interactions that we observed in our experiments. One type of horizontal cell (H1)^15,23,24,32^, connects cones to rods in the outer retina. This cell type exists in multiple species, including humans and mice. In the latter, targeted manipulation by use of a Gja10 promoter has already been achieved^12,23^, making it a favorable candidate for a therapeutic strategy. Therefore, we investigated whether H1 horizontal cells could be a potential therapeutic target to improve vision (Figs. 4a and 5a). We aimed to express the chemogenetic hyperpolarizing actuator hM4Di^33^ in horizontal cells, to mimic the hyperpolarizing input they receive from cones before photoreceptor degeneration. We used a gain of function approach to test whether this strategy would indeed provide an improvement in vision. For this, we performed experiments in *Cnga3*^*-/-*^ mice^34^, which lack cone function. Vision in these mice is mediated by rods and the absence of cone input deprives the rods of cone-driven H1 lateral feedback. This makes it an ideal model to confirm the nature of H1 feedback by gain of function studies (Fig. 1f). Notably, *Cnga3*^−*/−*^ mice^34^ show reduced contrast sensitivity (Fig. 4b and Supplementary Fig. 12), consistent with the saturation of rods in absence of cones and with our data from achromat patients (Figs. 2d and 3b). We identified a ~3 kb region within the *Gja10* promoter^4,12,23,35,36^ and successfully transduced horizontal cells using an AAV2/8 vector system (Fig. 4c, d; average transduction 29 ± 11%). We found that chemogenetic-driven hyperpolarization of H1 cells improved voltage modulation in the mouse outer retina, driven by photoreceptors and bipolar cells (Fig. 4e, f) as well as visual function (Fig. 4g). These results show that reactivation of lateral horizontal cell feedback can improve vision, consistent with these cells providing a lateral gain control input.

### Restoring horizontal cell feedback improves vision in a mouse model of geographic atrophy

Having established the effect on rod photoreceptors, we used a model of focal photoreceptor loss to evaluate this gain of function approach in a more therapeutically relevant scenario, where vision is mediated by cones + rods. Following macular degeneration, the region with a scotoma would deprive adjacent areas of lateral gain control input (Fig. 5a). Direct stimulation of horizontal cells in this circumstance could provide a therapeutic effect by restoring lateral input gain control, preventing rod photoreceptor saturation, and hence improving vision in these adjacent spared regions. To test our therapeutic strategy in these conditions, we wanted to measure the effect of horizontal cell activation in retinas with a focal loss of photoreceptor function and where remaining vision is mediated by both rods and cones.

To do this, we caused local scotomas in wild-type mouse retinas by laser ablation of RPE cells. We targeted the dorso-temporal retina since this region has been shown to have the highest density of retinal ganglion cells^37,38^ (Fig. 5b). Consistent with visual acuity depending on ganglion cell density^39^, lesions in this area caused a reduction in spatial acuity thresholds, that correlated with lesion size (Fig. 5c, d). Subretinal injection of hM4Di centered on the lesion led to strong transduction of horizontal cells (Fig. 5e–h). We then directly tested whether horizontal cell reactivation can improve remaining vision. We found that eyes expressing hM4Di mediated higher acuity vision following administration of the CNO activator (Fig. 5i, right panel). Instead, testing lesioned but untreated eyes showed no significant improvement in visual acuity thresholds (Fig. 5i, left panel). These results demonstrate that direct activation of horizontal cells is a viable strategy to improve vision in parts of the retina beyond the area of focal photoreceptor degeneration.

## Discussion

In this study, we investigated the importance of lateral feedback in modulating visual perception. Using psychophysical measurements in normal vision subjects we found that luminance changes control the gain of laterally displaced cells, substantially altering sensitivity to contrast and acuity thresholds. In particular, we investigated the effect of a scotoma on this lateral gain control feedback. Artificially removing some of the lateral feedback, using virtual scotomas during the presentation of test stimuli, significantly worsened visual function. In patients with a genetic form of macular degeneration^28^, loss of lateral input due to geographic atrophy limited remaining visual function in a similar way. Together, these results suggest that a scotoma not only deprives a focal spot on the retina of its function, but it also affects the surrounding spared retina by depriving a wider area of lateral feedback. The effect of lateral feedback has a wide reach, which is consistent with the long axons and extensive gap junction-mediated networks of laterally-projecting cells^4,12–15,35^. This contributes to a wide functional scotoma, which provides an opportunity for therapeutic intervention. Our experiments suggest that this impairment beyond the lesion may be an important and underappreciated aspect of the vision deficit in these and similarly affected patients.

We recruited patients with a range of photoreceptor dysfunctions in order to identify which photoreceptors benefit from and/or can drive this lateral feedback. By combining silent substitution methods^29^ with naturally occurring functional knock-out conditions, we found that lateral feedback optimizes both cone-mediated and rod-mediated vision. The observation for rod-mediated vision is of particular interest as it demonstrates that rod-mediated vision is not suppressed but improved by cone-driven feedback. As rod photoreceptors do not possess a dedicated pathway to the brain, activation of rods at mesopic light levels would affect cone signaling^4^. Although it has been suggested that a response suppression feedback from cones could resolve this problem^4^, our results show that rod function is optimized at mesopic light levels by a cone-driven gain control mechanism. Consistent with this, we found that patients lacking rod function have slightly worse contrast sensitivity than normal cone + rod-mediated vision, further indicating that rods make a positive contribution to vision in daylight conditions. By using rod-isolating stimuli, we observed that rod-mediated vision in patients where cone function remains (BED) is better than in patients without cone function (achromatopsia). These experiments also suggest that lateral gain control is driven predominantly by L/M cones and not by rods. It is possible that S-cones may also drive this lateral gain control, but their sparse distribution may limit their contribution.

Although several classes of laterally-projecting cells may be involved in the effect we observe^2,3^, several features make H1 horizontal cells a good candidate for gain of function experiments (see Results). To investigate the role of this cell in modulating rod function we used an exogenous actuator to perform gain of function studies in Cnga3^−/−^ mice^34^. Vision in these mice is mediated by rods only, which receive input from H1 horizontal cells. This provides an opportunity to observe directly the effect of horizontal cell activation, without possible contamination from changes in cone signaling. We found that activation of H1 cells was able to improve vision. Importantly, our results show that rod activity in absence of cones does not reflect normal rod function in daylight but rather that of rods, which, lacking lateral gain control, are poorly adapted and therefore poorly modulated by light stimuli (Fig. 3b). For this reason, loss of cone input to H1 cells offers a plausible mechanistic explanation as to how local or global loss of cone function deprives rods of an important gain control input and hence causes poorer vision and light aversion^40^. In Cnga3^−/−^ mice, rods can provide input to H1 cells via their gap junction connections with neighboring cones^35^. However, this input differs substantially from a cone-driven input and will be unable to give appropriate gain control at mesopic/photopic light levels. Our results in human subjects suggest that a focal scotoma, which removes only part of the lateral feedback, significantly alters the remaining cone + rod vision. We found the same to be true in a mouse model of focal photoreceptor loss and were able to partially reverse this effect by chemogenetically mimicking light input to H1 cells.

Several features of this strategy make it a good candidate for translation to the clinic. Horizontal cells that have lost the overlying photoreceptors might be more easily targeted by subretinal injections, as indicated by the higher transduction we observed in lesioned mice as opposed to mice with an intact photoreceptor layer (cf. Fig. 4d vs Fig. 5g). Also, we find an effect of lateral feedback over several degrees of field of view (Supplementary Fig. 13). Notably, ~30% of patients with AMD have a progression of geographic atrophy between a third and half of a degree per year^41^. Together, these findings indicate that our strategy could provide an improvement in vision in these patients for over a decade of life. In addition to improving remaining vision^42^, lateral feedback may also slow degeneration of the surviving photoreceptors, by preventing saturation of rods. Indeed, in models where cone input and H1 lateral feedback is absent, rod photoreceptors numbers tend to decline^12,43^. In summary, our findings advance the understanding of the pathology of macular degeneration. We also provide evidence in mice for a potential therapeutic strategy to improve vision following focal photoreceptor loss.

## Methods

### Psychophysics experiments on human subjects

This study adhered to the tenets of the Declaration of Helsinki and was approved by the Moorfields Eye Hospital Ethics Committee (NHS REC reference: 11/H0703/10). Informed consent was obtained from subjects or consent and assent were obtained from parents and children, respectively, prior to entering the study. Patients consented to their medical notes may be looked at by the research team, that clinical images and information be published in scientific journals, and to have tests of the retinal function be performed. No compensation was provided to the participants but travel costs were reimbursed for some patients. All psychophysical tests involved a two-alternative forced-choice task to detect the presence of stimuli on the left or right of a central fixation point. A chin/head-rest maintained head position and viewing distance during the tasks. For a subset of subjects, comparative measurements were made with and without the aid of an eye-tracking device (Tobii Pro, version X-120, Tobii SDK3.0, acquisition via Matlab R2014b). For these experiments, a calibration procedure was performed at the start of the session for each subject. The gaze outlier criterion was set at 100 pixels. The similarity between measurements made with and without pupil tracking suggested that pupil tracking did not significantly alter contrast threshold measurements and was therefore not used to generate the data presented in this study. This comparative data is presented in Supplementary Fig. 4. Stimuli were presented via a gamma-corrected CRT monitor, placed 0.85 m from the chin rest. Screen resolution was set at 1024 × 768 pixels. The screen size was 30.5 cm × 40 cm. All tasks were performed binocularly, except for experiments in Stargardt patients for which the left eye was patched. Stimuli were generated in Matlab (Matlab R2014b, MathWorks Inc, Natick, MA, USA) and delivered to the CRT monitor via a ViSaGe MKII Stimulus Generator, (Library version 8.201, VSG DLL version 1.271, toolbox version 1.271, Cambridge Research Systems Ltd, Rochester, UK). Contrast sensitivity thresholds were determined using a QUEST algorithm procedure (Watson and Pelli, 1983). All tests used a fixed number of reversals (a minimum of 14 and a maximum of 28) to estimate a subject’s threshold. Stimuli were presented for 2 s and subjects were allowed to respond without a timeout limit. Throughout the manuscript threshold values have been presented as contrast sensitivity (calculated as the inverse of the estimated threshold value). Normal vision subjects included 12 males and 10 females, aged 18–52. Three subjects (authors M.R., K.P., and A.G.) completed all tests. All other subjects completed a subset of tests. Subjects had to fixate a fixation spot placed in the center of the monitor. Stimuli were presented at the same elevation as the fixation spot and at the specified and identical left or right eccentricity. Presentation either left or right was randomized and subjects were asked to report where they perceived the stimulus by pressing a left or right button on a console. For Stargardt patients, the location of the stimulus presentation was chosen on the basis of microperimetry data (see below). For the majority of experiments, the silent substitution technique (Estevez et al. 1982) was used to exclude s-cone activation. The rationale for this method was to negate possible differences arising from previously reported anatomical isolation of the s-cone pathway from interaction with rod input. All tests in patients with disrupted cone function (achromatopsia and blue cone monochromacy) were performed with non-selective stimuli modulated along a black-to-white axis. Comparative tests using black-to-white stimuli in normal vision subjects confirmed the main finding. Temporally modulated stimuli were presented as Michelson contrast sinusoids. A 0.2 s ramp was used at the beginning and the end of the stimulus. Gabor patches were used for spatially modulated stimuli. The Gabor’s sigma was set to 0.5 degrees and the annulus surrounding the Gabor stimulus had an aperture of 1-degree diameter through which the stimulus was visible. The range of contrasts presented varied between 0.1 and 30%. No measured threshold reached these minimum or maximum values. Patients with Bornholm eye disease only express three types of opsin in the retina (in two of the patients tested, the expression was S-/M-/Rhodopsin and in the third, S-/L-/Rhodopsin). As our CRT monitor enables isolation of up to three types of opsin we were able to generate rod photoreceptor-isolating stimuli for these patients, allowing us to specifically activate rods with our test stimulus. Surround annuli that contained more than one luminance were calibrated in order to have the same area dedicated to each luminance. For all annuli, the width was defined as the difference between the outer and inner radius of the annulus.

A typical testing session with a single subject would normally allow measurement of 6–7 contrast sensitivity thresholds. No subject was tested for more than 1 h in a single session. If a session consisted of testing a range of experimental values (for example, a range of surround luminance values or stimulus frequency values) these were presented to the subject in pseudo-random order. Temporal contrast thresholds were measured using sinusoidally modulated stimuli at a range of cycle frequencies to build contrast sensitivity functions. Single measurement tests were performed at a 4 Hz cycle frequency. Spatial contrast thresholds were measured by presenting a Gabor patch with a range of fixed spatial frequencies, at varying contrasts. Conversely, spatial acuity measurements were performed by presenting a 40% contrast Gabor patch at varying spatial frequencies. In a small set of experiments, the surround changed only during the presentation of the stimulus in the center (in order to test for/avoid adaptation to the mismatched surround). In this case, the surround was equiluminant until the stimulus was presented and returned to equiluminant once the choice was made and the trial ended. Data from two subjects in which measurements were made with these presentation parameters is presented in Supplementary Fig. 3.

### Microperimetry and fundus autofluorescence imaging in Stargardt disease patients

Microperimetry was performed monocularly using the Nidek MP-1 (Software version 1.7.8, Nidek Technologies, Padova, Italy). Pupils were dilated and cyclopleged using 2.5% phenylephrine hydrochloride solution and 1% tropicamide ophthalmic solution. Before testing, the Spectralis OCT (Software version 6.9a, Heidelberg Engineering, Heidelberg, Germany) was used to obtain a single transfoveal horizontal line scan. This was imported and used by the Nidek MP-1 manufacturer’s software as an aid to automatically locate the anatomic fovea to facilitate accurate foveal placement of the testing grid. Fixation stability was determined prior to commencing microperimetry testing by sampling at 25 Hz for 5–15 s. During the microperimetry exam, the tracking continuously traces (as before) the position of the patient’s fixation in time: this information is used to precisely correct the stimuli projection locations so as to compensate for eye movements during the exam. If tracking is lost, the exam is suspended and no stimuli are presented until tracking can resume. Testing consisted of a 4 apostilbs (1.27 cd/m2) background, Goldmann size III stimulus presented for a duration of 200 ms, and a 4 to 2 dB full-threshold bracketing test strategy. The customized testing grid consisted of 44 testing locations (see Fig. 1i, Supplementary Fig. 7 and also Tanna et al., 2018). The grid pattern was of radial design with centrally-condensed spacing and covered the macular and paramacular regions. All tests were performed under almost dark (mesopic) light conditions. The sensitivity at each retinal location was determined by iteratively adjusting the light intensity until the dimmest visible stimulus was found. The sensitivity for each test location was determined on a scale of 0 to 20 dB (MP-1 scale), with higher values indicating greater sensitivity. Only right eye data were used. Results from the microperimetry were used to estimate the functional border of the scotoma for each Stargardt patient. Test locations with 0–1 dB scores (i.e., retinal locations where only the brightest stimulus was detected or no stimulus at all was detected) were defined as scotoma, and stimuli were placed laterally adjacent to these locations for psychophysical testing. Before commencing the QUEST estimation of contrast sensitivity, we confirmed the suitability of the location by empirically testing the stimuli locations; patients were asked if they could see a 1-degree test stimulus (a static gray circle on a black background). If necessary, the test stimulus was moved laterally until the patient reported being able to clearly see it (location as shown in the figures). Fundus images were acquired by white light reflectance imaging. Short wavelength (486 nm) fundus autofluorescence was obtained using a Heidelberg Spectralis Scanning Light Ophthalmoscope.

### Gain control model fits

The data fits shown in Supplementary Fig. 6 were obtained by fitting a third-order polynomial to each data set (stimuli presented within the same surround). To test whether the effect of surround luminance is better described by an input gain control or a response gain control model, the fit obtained for one of these data sets (75% surround, *f(x)* = 0.33*x*3-1.36*x*^2^ + 2.11*x* + 0.02) was divided by the ratio between this and all other luminance values. For the input gain control model, the divisive factor was applied to the independent variable in the polynomial equation. For the response gain control model the divisive factor was applied to the dependent variable. Model fits were generated in Python (version 3.7) and, in their final version, using Matlab (R2018a, MathWorks Inc, Natick, MA, USA).

### Experiments in mice

All experiments in mouse were approved by the local Animal Welfare and Ethics Review Board (UCL, London, UK) and the Home Office and conformed to the guidelines on the care and use of animals adopted by the Society for Neuroscience and the Association for Research in Vision and Ophthalmology (Rockville, MD, USA). Experiments were performed under project license no. PCCD291B7. Mice were housed with a 12 h/12 h light/dark cycle and standardized environmental enrichment (nesting materials, Perspex play tunnels). Holding rooms and procedure rooms were maintained between 19–21 °C and humidity between 45–65%. C57BL/6 mice were obtained from Harlan, UK. *Cnga3*^*cpfl5/cpfl5*^ mice^34^ (referred to in the manuscript as *Cnga3*^*−/−*^) were obtained from J.R. Heckenlively (University of Michigan).

### AAV vector production and testing

A 3.0 kb sequence from the mouse Gja10 promoter was amplified by PCR from mouse genomic DNA and cloned into an AAV viral vector containing the gene for Cre recombinase. Recombinant AAV vectors were produced via triple transient transfection. The plasmid construct, AAV serotype-specific packaging plasmid, and helper plasmid were mixed in a ratio of 1:1:3 at 20 µg total DNA per ml of DMEM, were mixed with Polyethylenimine (Polysciences Inc.) to a final concentration of 50 mg/mL and incubated for 10 min at room temperature to form transfection complexes. These were added to HEK293T cells at 50 µg DNA per 175cm2 and left for 72 h. The cells were collected, concentrated, and lysed by freeze-thaw (4x) in TD buffer to release the vector. AAV particles were purified by affinity with an AVB Sepharose column (GE Healthcare) and eluted with 50 mM Glycine pH2.7 into 1 M Tris pH 8.8. AAV2/9 was purified by a combination of size exclusion and anion exchange. Sephacryl S300 and Poros HQ50 respectively (GE Healthcare). Vectors were washed in 1x PBS-MK and concentrated to a volume of 100–150 µl using Vivaspin 4 (10 kDa) concentrators. Vector genome (vg) based titers were determined by quantitative real-time PCR (qPCR) using an ITR binding assay. Constructs encoding for the backbone and the subretinal injections were performed in adult wild-type and *Cnga3*^*−/*−^ mice. Tests for vector specificity were performed by subretinal injection in Ai9 (lox-STOP-lox-tdTomato, Allen Institute) mice. Two injections were performed in each eye (in the superior and inferior hemispheres respectively). Two microliters of virus (titer 1 × 10^13^ vg/ml) were injected. When two viruses (pAAV-Gja10-Cre and pAAV-lox-STOP-lox-hM4Di) were co-injected, equal volumes of 2 × 10^13^ vg/ml were mixed in a vial prior to injection. The virus was diluted following purification and to the appropriate titer in sterile PBS-MK. Control injections were performed with the same volume of PBS-MK. For each mouse, one eye was randomly assigned virus injection and the other sham injection. All mice were allowed to recover for at least 3 weeks following subretinal injections before behavioral testing. Quantification of horizontal cell transduction was performed by co-staining with a Calbindin D-28k antibody (CB-38, Swant, dilution 1:500, validated for horizontal cells in Burger et al., 2020). Quantification of ganglion cell density was performed by staining with an anti-Brn3a antibody (Santa Cruz Biotechnology, dilution 1:500, sc-31984, C-20, previously validated for retinal ganglion cells by Nadal-Nicolas et al., 2009; Nadal-Nicolas et al., 2010; Nadal-Nicolas et al., 2012).

### Mouse optomotor behavior

Mice were tested for Optomotor reflex in a setup (Cerebral Dynamics, NY, USA) consisting of four computer screens, simulating a rotating drum. An experimenter blind to the content of the subretinal injection performed scoring of the behavior. A clockwise vs counterclockwise two-alternative forced-choice was used. The threshold was determined once the test completed seven reversals within 1% contrast. All acuity tests were performed at a fixed 100% contrast. Contrast sensitivity tests were performed at the indicated spatial frequencies. Mice were allowed to adapt to the set-up platform for 10 min on the first day before testing began. All tests were repeated four times for each condition for each mouse. In order to allow activation of the chemogenetic tool hM4Di, mice were intraperitoneally injected with a Clozapine-N-oxide compound between 1 and 3 h before testing took place. Twenty-four hours were allowed to pass before the following test was performed, in order to allow termination of CNO-mediated activation of the exogenous receptor. Either spatial acuity or contrast sensitivity thresholds were measured.

Optomotor tests with a thin stimulus were performed in the same setup (with the stimulus presented on all four screens). The stimulus was generated by a custom Matlab script and consisted of a left- or right-ward drifting sinusoidal, placed in the center of the screen. A QUEST Staircase procedure was used to determine the contrasts to be presented to the mouse. The surround (consisting of the entire screen except for the 1-degree height stimulus) was either black, gray (coinciding with the midpoint of the sinusoidal stimulus), or white. To avoid adaptation to a different light level, all screens were gray during inter-trial intervals and the surround was only presented simultaneously with the drifting stimulus.

### Lesion studies

Laser lesions of RPE cells were carried out with a Microlase laser. A fundus imaging camera aided the placement of lesions in the dorsal-temporal quadrant of the retina, where the density of retinal ganglion cells is maximum. Six to eight laser pulses were delivered to each eye in one session (spot diameter 500 um, power 60 mW, duration 200 ms). The animals were anesthetized with an intraperitoneal injection of a 0.007 ml/g mixture of medetomidine hydrochloride (1 mg/ml), ketamine (100 mg/ml), and water at a ratio of 5:3:42 before recording. Pupils were dilated with a Tropicamide/Phenylephrine mix. Immediately after the lesion, a subretinal injection was performed in either the left or right eye (chosen by a person who had not performed the lesions). The other eye remained uninjected. Mice were allowed to fully recover for at least 3 weeks before undergoing optomotor testing. The injection volume was 0.4 ul.

### Fundus imaging

Six to eight weeks post-lesion/injection, fundus imaging was performed on mice that had not been culled at an earlier point and had not developed a cataract during anesthesia. Eyes were imaged with a Micron III imaging platform (Phoenix Research Laboratories). Images were taken with the same objective with and without a GFP filter. Animals were anesthetized with an intraperitoneal injection of a 0.007 ml/g mixture of medetomidine hydrochloride (1 mg/ml), ketamine (100 mg/ml), and water at a ratio of 5:3:42 before recording. Lesion size was estimated by a person blind to the results of behavioral experiments, by tracing contours of lesions and measuring resulting areas with ImageJ (ImageJ1.2a).

### Histological analysis

Eyes for histological analysis were taken post-mortem. Retinas were extracted and fixed in 4% PFA overnight. A histological clearing procedure (adapted from Costantini et al., 2015) was performed on whole retinas to maximize imaging quality and aid verification of expression of the fluorescent protein in Ai9 mice and mice co-injected with pAAV-Gja10-Cre and pAAV-EF1A-DIO-hM4Di-EGFP. To estimate densities, retinas were whole-mounted. A custom MATLAB script was used to count horizontal cells while looking at the retinas under the microscope. Three locations were counted within the lesion area (identified by lack of photoreceptor layer over the horizontal cells) and three locations 1 mm from the edge of the zone with no photoreceptors. Density was estimated per mm^2^.

### Electroretinogram (ERG)

The animals were anesthetized with an intraperitoneal injection of a 0.007 ml/g mixture of medetomidine hydrochloride (1 mg/ml), ketamine (100 mg/ml), and water at a ratio of 5:3:42 before recording. Pupils were fully dilated using 1.0% tropicamide. The subdermal ground was inserted in the mouse’s left cheek. A drop of Viscotears 0.2% liquid gel (Dr. Robert Winzer Pharma/OPD Laboratories, Watford, UK) was placed between the electrode and the eye. Bandpass filter cutoff frequencies were 0.312 and 1000 Hz. Flicker series were performed at 0.1 cd/m^2^ light intensity as previously reported (Nishiguchi et al., 2015). The frequencies tested were 1, 3, 6, 9, 12, and 15 Hz. Three repetitions were performed before and three after CNO injection. Mice were moved and repositioned after every repeat. Tests before and after CNO injection were performed on different days, as the induction time (1–3 h) would not be covered by the same injection of an anesthetic.

### Statistics and reproducibility

Unless otherwise stated averaged data is represented as mean ± standard deviation. The statistical tests used for each experiment are stated in the corresponding figure legend and in Supplementary Table 1. The experiments presented in Fig. 1i, j were performed once for every patient visit, which occurred roughly every 6–12 months, for at least two visits. Similar results were obtained across visits. Images similar to the representative example shown in Figs. 4d and 5g were obtained in ten mice (13 images total) and 11 mice (11 images total), respectively.

### Reporting summary

Further information on research design is available in the Nature Research Reporting Summary linked to this article.

## Supplementary information

Supplementary Information

Reporting Summary

Peer Review File

## Data Availability

Source data are provided with this paper. The data generated in this study are available in the Supplementary information/Source Data file. Source data are provided with this paper.

## Source data

Source data

## Code availability

The code used to program and present the visual stimuli presented in this study are available in a public database.

https://github.com/matteorz/Rizzi_Powell_et_al_2022
