# Supplementary material for: Lateral gain is impaired in macular degeneration and can be targeted to restore vision in mice
Source: Nat Commun. 2022 Apr 20;13:2159. doi: 10.1038/s41467-022-29666-x (PMC9021237; doi:10.1038/s41467-022-29666-x)
Supplement: Supplementary file 1 — Supplementary Information [file 41467_2022_29666_MOESM1_ESM.pdf]

Supplementary figure 1

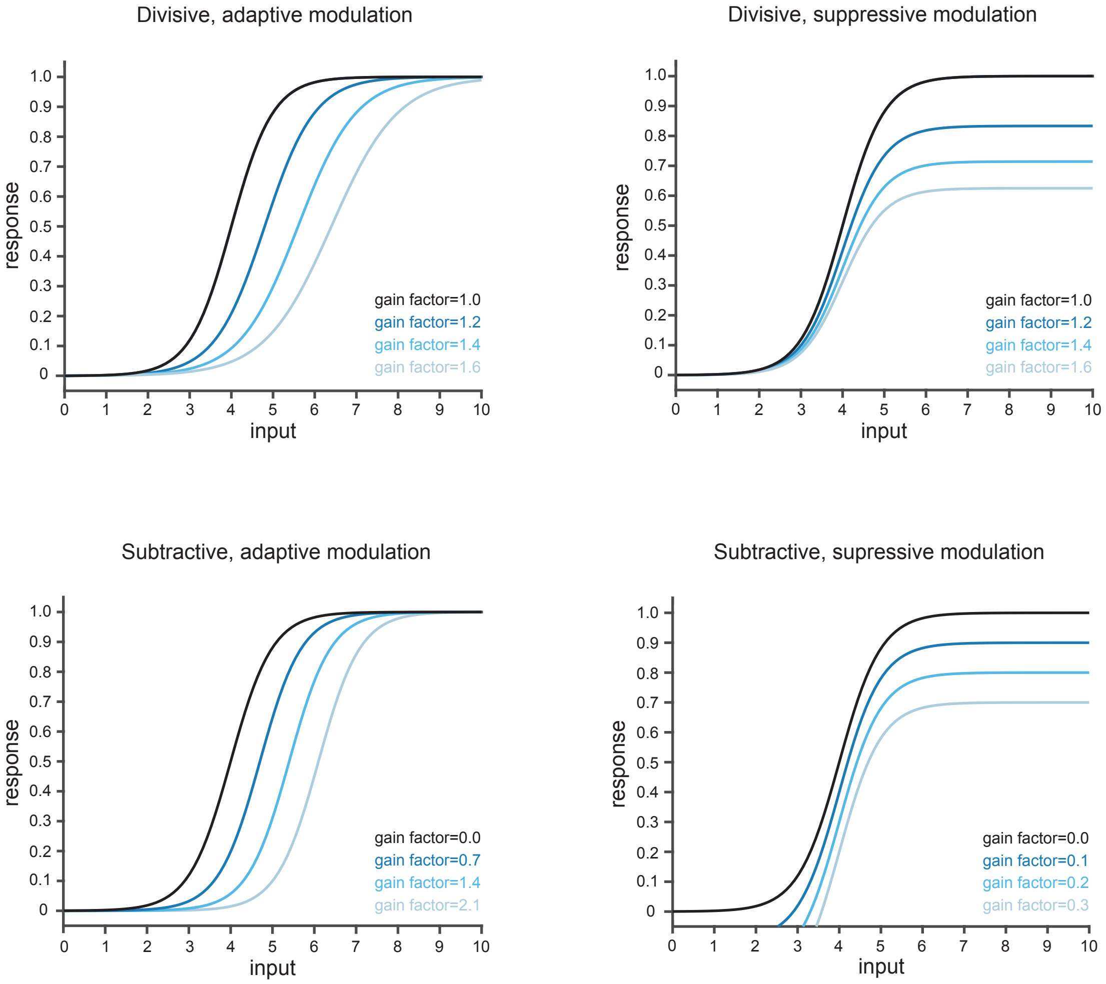

Mathematical models of different feedback mechanisms showing how they modulate the input-response relationship.
Schematic showing input-output functions at different gain values (indicated by corresponding color), when the gain is adaptive, implemented through a division of, or a subtraction from the input (left top and left bottom panels respectively) or suppressive, by the division of or subtraction from the response (right top and right bottom panels respectively). When the gain factor is applied to the input the shift/scale occurs along the x-axis (left panels), and when applied to the response it occurs along the y-axis (right panels). Implementing the gain through a divisive mechanism (top panels) scales the function, whereas implementation through a subtractive mechanism shifts the function without scaling (bottom panels). Note that different gain factor values were applied for the different computations for illustration purposes.

Supplementary figure 2

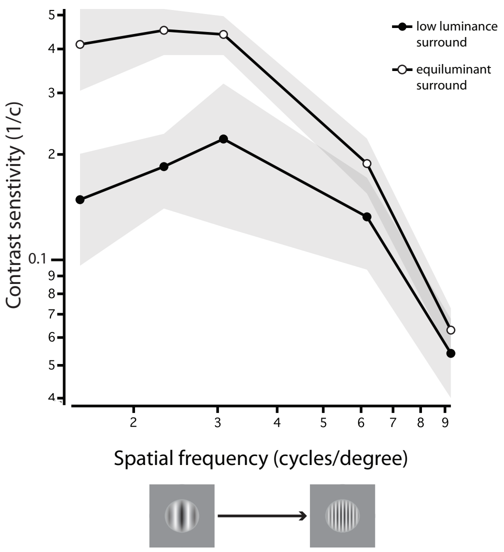

Surround luminance affects spatial contrast sensitivity functions in normal vision subjects.
An equiluminant surround improves spatial contrast sensitivity functions. Gabor patches of different spatial frequencies were presented inside a 1° stimulus area, surrounded by a 1.75° annulus width (n=9 subjects; 0.05 cycles/deg, p=0.0057; 0.075 cycles/deg, p=<0.0001; 0.1 cycles/deg, p=0.0053; 0.2 cycles/deg, p=0.010; 0.3 cycles/deg, p=0.31. All *p*-values were calculated using a one-way ANOVA, with Tukey's post hoc multiple comparison test). All contrast sensitivity values are calculated as 1/contrast threshold (1/c). Values shown are mean averages and shaded regions denote standard deviations. Source data are provided as a Source Data file.

Supplementary figure 3

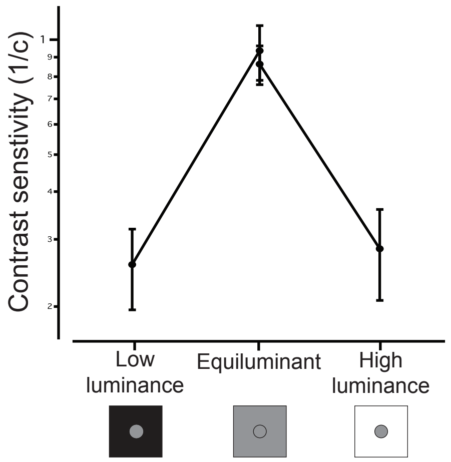

Surround luminance acts as an adaptive mechanism
A high luminance (64 cd/m^2) surround reduced contrast sensitivity similarly to a low luminance surround (n=9 normal vision subjects). Error bars denote standard deviation around a mean value. Source data are provided as a Source Data file.

Supplementary figure 4

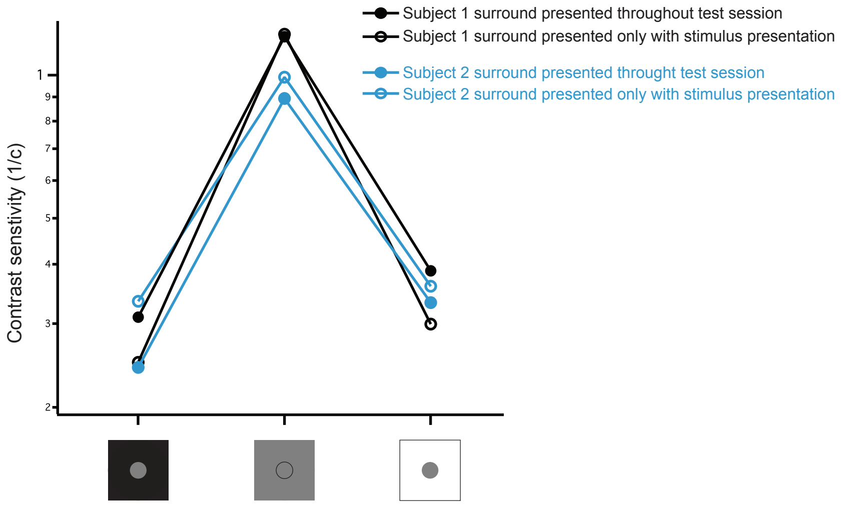

Surround-mediated effects do not require prolonged adaptation to surround luminance.

Comparison of temporal contrast sensitivity with low luminance, equiluminant and high luminance surrounds in two normal vision subjects, each indicated with a different color. Contrast thresholds were measured using a 1° stimulus placed at a 6° eccentricity, with a 4 Hz temporal sinusoidal modulation. Closed circles indicate measurements made with the luminance of the background set throughout the test session. Very similar measurements were obtained (open circles) when the background luminance was set to equiluminant during the response period between stimuli presentations and only decreased (to black) or increased (to white) to coincide with stimulus onset. The similarity between the measurements obtained with these two different presentation parameters indicates that the effect of luminance in the surround is not due to slow adaptive mechanisms and that the surround provides fast feedback to the center. Source data are provided as a Source Data file.

Supplementary figure 5

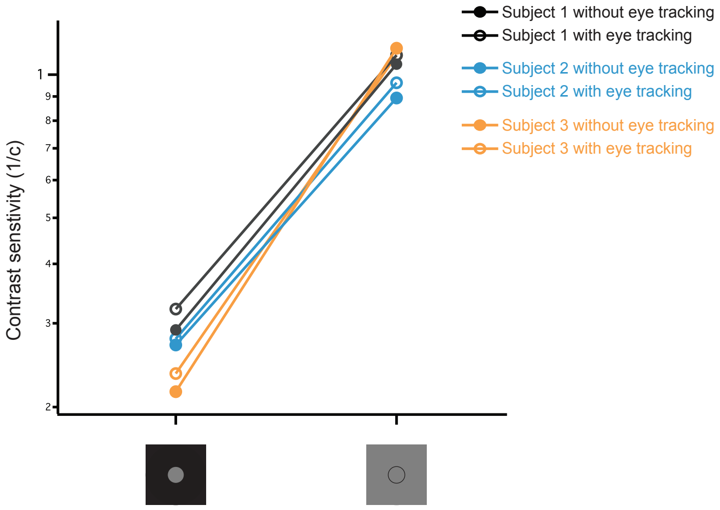

Comparable contrast threshold measurements obtained with and without eye tracking.

Temporal contrast sensitivity in three normal vision subjects each indicated with a different color. Subjects were asked to fixate a spot placed in the center of the CRT monitor. Stimuli were 1 degree diameter, 4 Hz sinusoidal temporal modulation presented at 6° eccentricity on either side of the fixation spot. Subjects were asked to indicate on which side the stimulus was presented. Their ability to detect the stimulus was determined by their forced choice response. Contrast sensitivity was reduced by a low luminance (0.15 cd/m^2) vs luminance matched (27.7 cd/m^2) surround. Closed circles indicate threshold measurements made without the aid of a pupil-tracking device. Very similar threshold values were obtained (open circles) when measurements were made with the aid of a pupil tracking device that automatically discounted trials if saccades were made during the presentation of the stimulus (see Methods). Source data are provided as a Source Data file

Supplementary figure 7

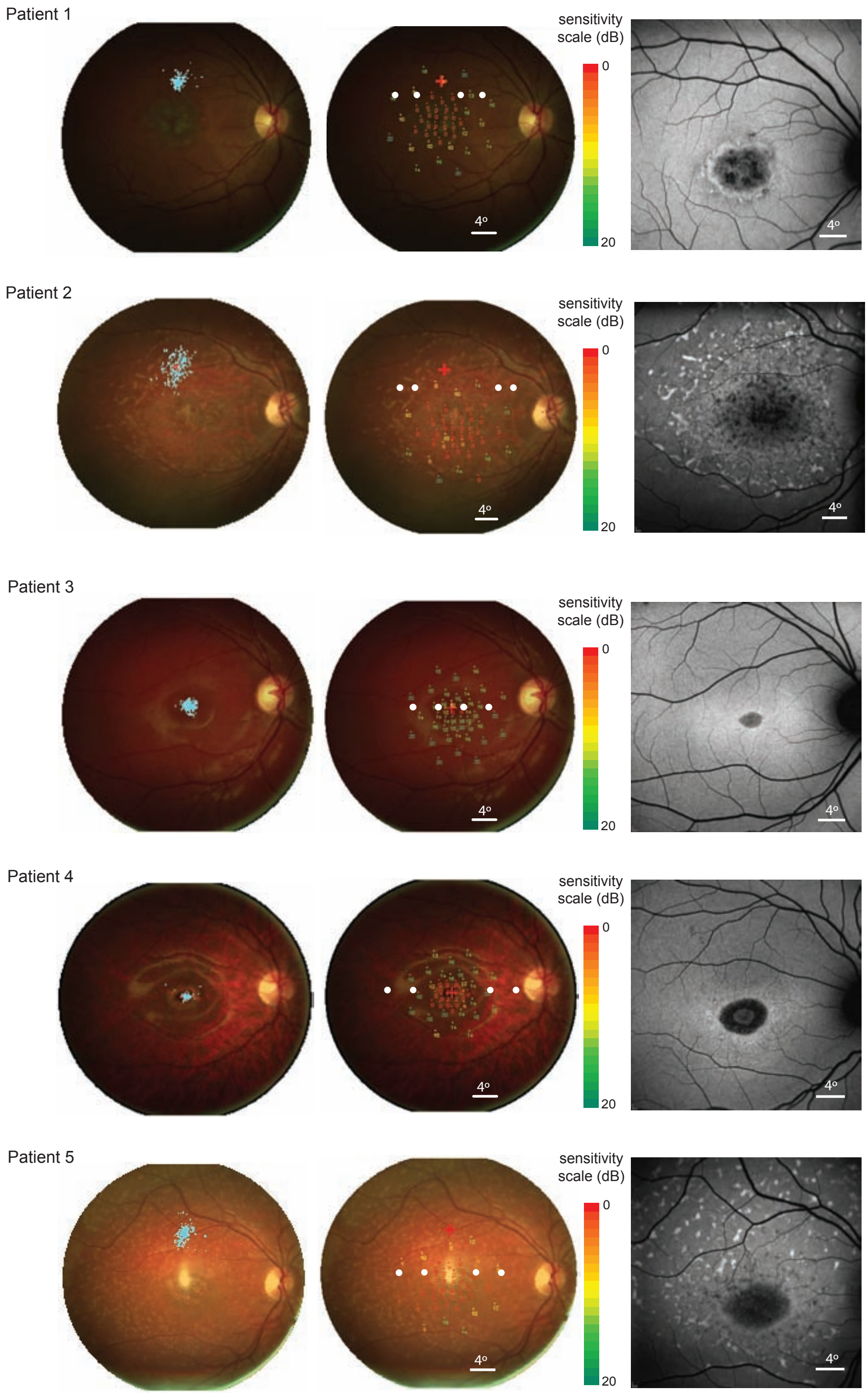

Microperimetry data and Fundus Autofluorescence images for all tested Stargardt disease patients.

Left column: fixation stability (in cyan, see Methods) superimposed on a color fundus image of the eye. Middle column: sensitivity values obtained from microperimetry superimposed on color fundus photograph of the eye. Color bar indicates sensitivity in decibels. Red cross indicates average fixation spot for images in left and middle column. Superimposed white circles indicate stimulus size and locations used for psychophysical testing of contrast sensitivity proximal (central placement) and distal (peripheral placement) to the lesion. Right column: short wavelength (486 nm) Fundus Autofluorescence images. The area of atrophy presents with a decreased signal in all patients.

Supplementary figure 8

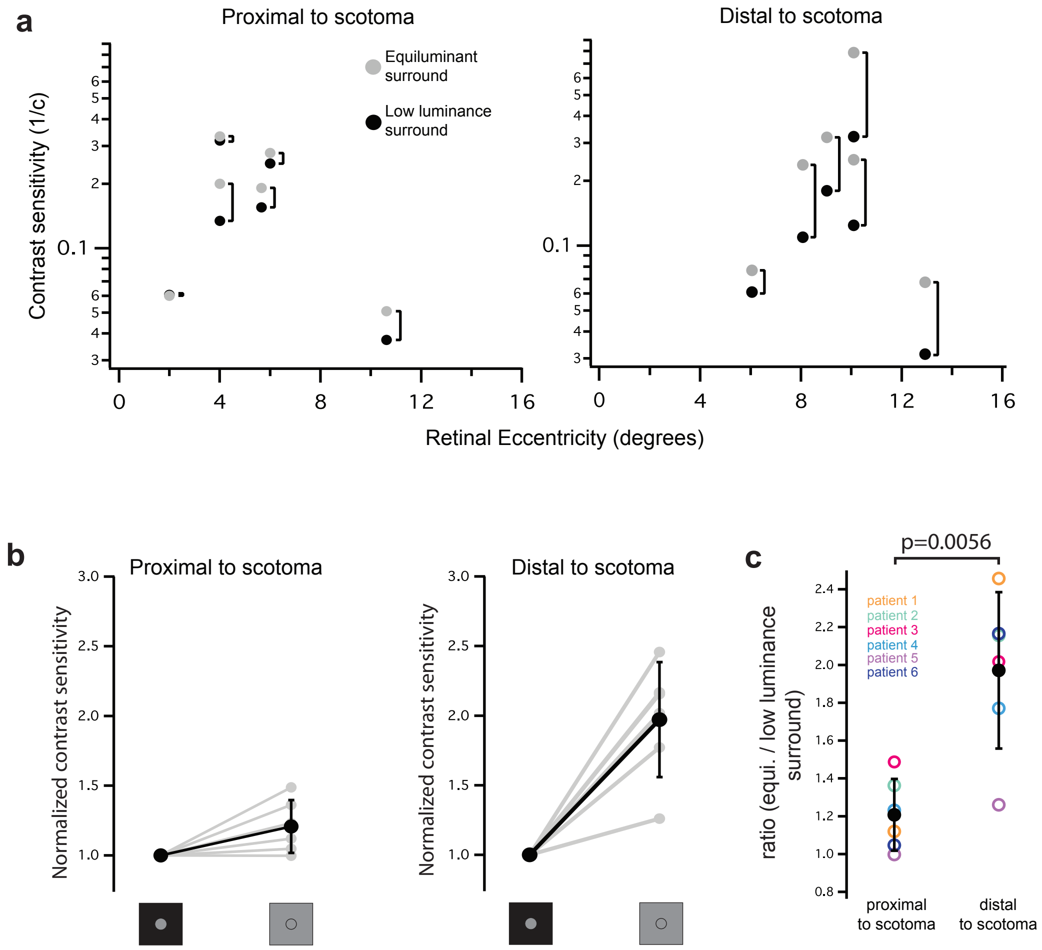

Patients with Stargardt disease do not have a generalized lack of lateral adaptation.

a, Paired measurements in each patient (n=6 patients) show that an equiluminant surround imposes an improvement in vision (indicated by brackets) in locations distant from the scotoma similar to that observed in subjects with normal vision (cfr. Fig.1g, no scotoma). Locations of measurements were determined by retinal function as indicated by microperimetry measurements for each patient (see Methods). **b**, Same data as shown in panel **a** normalized to low luminance measurements within each patient to allow comparison of the data across the different retinal locations and with Fig.1g (n=6 patients). **c**, Data shown in panel a, expressed as a ratio of contrast sensitivity for equiluminant over low luminance surrounds at proximal and distal locations. Data from each patient is indicated by a different color (n=6 patients, p=0.0056; two-sided paired t-test). Error bars denote standard deviations around mean values. Source data are provided as a Source Data file.

Supplementary figure 9

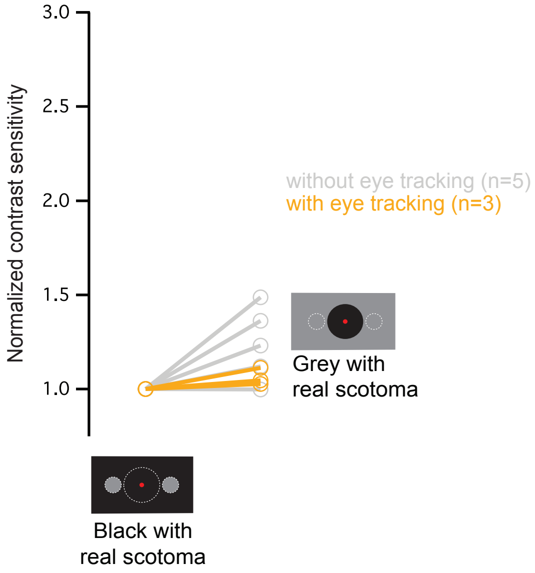

Comparison of contrast sensitivity measurements made with and without eye -tracking in patients with Stargardt disease.
Comparison of contrast thresholds in patients with Stargardt disease. Measurements were made at locations adjacent to the lesion, and when surrounded with low or equi-luminant surrounds (data normalized to contrast sensitivity measured with a low luminance surround). Measurements made without (grey, n=5 patients) or with (orange, n=3 patients) eye tracking were comparable, indicating that eye tracking did not greatly alter the accuracy of contrast sensitivity measurements. Source data are provided as a Source Data file.

Supplementary figure 10

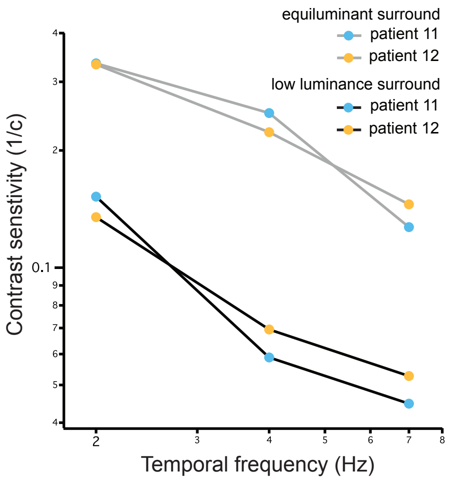

Rod-only temporal contrast sensitivity functions for two patients with Bornholm eye disease.
Silent substitution allowed selective stimulation of rod photoreceptors. For both patients, contrast sensitivity was worsened by a low luminance (black trace) vs equiluminant (grey trace) surround at all tested frequencies. (Data at 4hz also shown in Fig.2b,c). Source data are provided as a Source Data file.

Supplementary figure 11

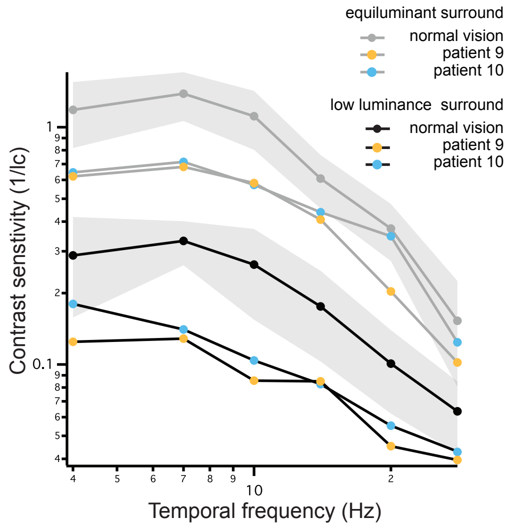

Temporal contrast sensitivity functions for two patients with Congenital Stationary Night Blindness

For two patients, contrast sensitivity was improved by an equiluminant (grey traces) vs low luminance (black traces) surround. Comparative data for normal vision subjects is shown (grey and black markers, n=8 normal vision subjects). Values shown are mean averages, standard deviation indicated by grey shading, data as shown in Fig.1b). Source data are provided as a Source Data file.

Supplementary figure 12

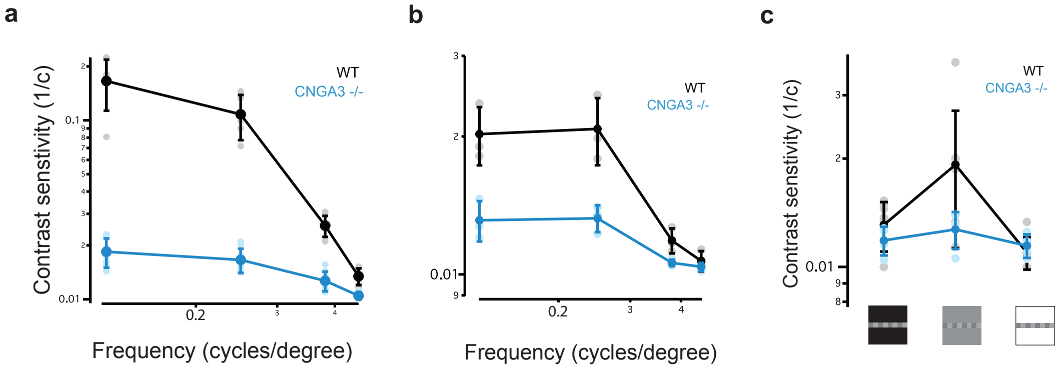

Reduced contrast sensitivity in Cnga3$^{-/-}$ mouse model of achromatopsia.
a, Contrast sensitivity was reduced in Cnga3$^{-/-}$ mice (n=9 mice) compared with wild-type mice (n=5 mice) measured using a standard optomotor behavior (0.128 cycles/degree p<0.0001; 0.25 cycles/degree p<0.0001; 0.381 cycles/degree, p=0.88, 0.45 cycles/degree, p=0.99; one-way ANOVA with Tukey's post hoc multiple comparison test). **b**, Contrast sensitivity functions could be obtained for wild-type (n=3 mice, black trace) and Cnga3$^{-/-}$ (n=3 mice, blue trace) mice using a modified optomotor behavior paradigm driven by a 1° height sinusoidal stimulus. As expected, contrast sensitivity values were reduced compared to those obtained with full screen presentation of the same stimuli (panel a). **c**, The modified optomotor method allowed for assessment of different surround luminance values on contrast encoding in mice. Wild-type mice (black trace, n=9 mice) showed increased contrast sensitivity with an equiluminant surround compared to low- and high-luminance conditions, whereas Cnga3$^{-/-}$ mice (blue trace, n=4 mice) did not. Note that data for low and equiluminant surrounds in panel c is also shown in Figure 4. Error bars denote standard deviations around mean values. Source data are provided as a Source Data file

Supplementary figure 13

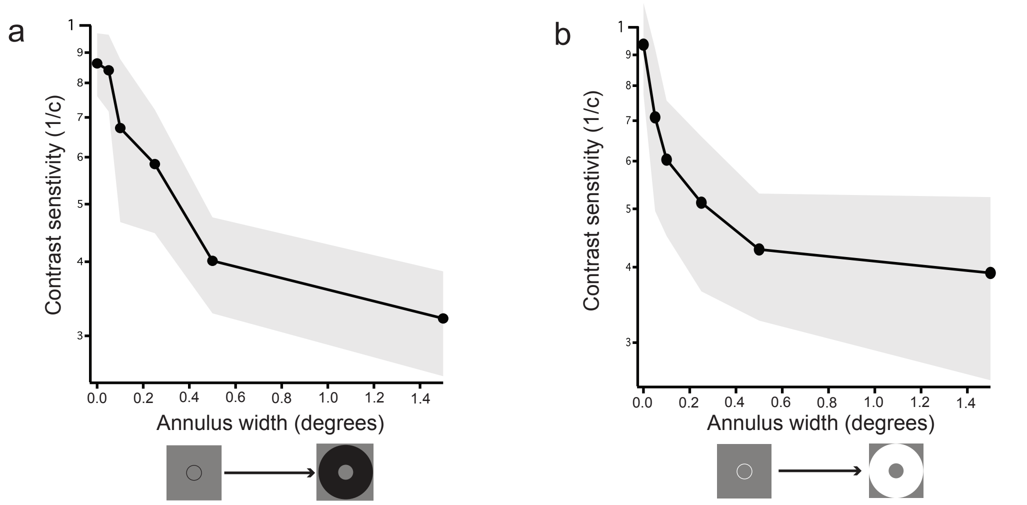

Low and high luminance surrounds degrade contrast encoding over a wide retinal area.

a, Expanding a black, low luminance (0.15 cd/m2) annulus around the 1° stimulus lead to a cumulative degradation of contrast encoding compared to the scenario of no surround. (n=9, Annulus width: 0.05 degree, p=0.98; 0.1 degree, p=0.08; 0.25 degree, p=0.0028; 0.5 degree, p<0.0001; 1.5 degree p<0.0001; one-way ANOVA, with Dunnett's post hoc multiple comparison test). This effect of the surround decays in proportion to the distance from the center. **b**, Surrounding the stimulus area with a white, high luminance (64 cd/m2) annulus reduced contrast sensitivity similarly to a low luminance annulus. The effect was also cumulative and a thin edge (0.05° annulus width) was not sufficient to reproduce the entire effect (n=9 normal vision subjects; Annulus widths: 0.05 degree, p=0.0013; 0.1 degree, p<0.0001; 0.25 degree, p<0.0001; 0.5 degree, p<0.0001; 1.5 degree p<0.0001; one-way ANOVA, with Dunnett's post hoc multiple comparison test). All contrast sensitivity values are calculated as 1/contrast threshold (1/c). Values shown are mean averages and shaded regions denote standard deviations. Source data are provided as a Source Data file

Statistical Summary Table

Fig.1b

One-way ANOVA with Tukey post hoc multiple comparison test
Comparing equiluminant to low luminance temporal contrast sensitivity for each tested frequency
95% CI

Comparison	P-value	Effect Size	95% CI	DF
4Hz equilluminant vs low luminace	0.0018	0.935	0.4156 to 1.454	7
7Hz equilluminant vs low luminace	0.0010	1.053	0.5227 to 1.582	7
10Hz equilluminant vs low luminace	0.0046	0.8463	0.2997 to 1.393	7
14Hz equilluminant vs low luminace	0.0043	0.4313	0.1558 to 0.7067	7
20Hz equilluminant vs low luminace	0.0009	0.2713	0.1361 to 0.4064	7
28Hz equilluminant vs low luminace	0.0550	0.08875	-0.001975 to 0.1795	7

Fig.1c

Two tailed paired t-test
Comparing equiluminant to low luminance spacial contrast sensitivity
95% CI

Comparison	P-value	Effect Size	95% CI	DF
equilluminant vs low luminace		-6.487	-8.476 to -4.497	5

Fig.4f

Two-way ANOVA with Sidak post hoc multiple comparison test
Comparing power of tested frequencies pre and post administration of CNO in treated and untreated eyes
95% CI

Comparison	P-value	Effect Size	95% CI	DF
1hz Untreated pre vs post CNO	0.9888	1022	-17622 to 19666	8
3hz Untreated pre vs post CNO	0.9982	-333.3	-15714 to 15047	8
6hz Untreated pre vs post CNO	0.9763	874.4	-10065 to 11813	8
9hz Untreated pre vs post CNO	0.3922	2694	-2517 to 7906	8
12hz Untreated pre vs post CNO	0.8729	254	-1074 to 1582	8
15hz Untreated pre vs post CNO	0.8729	254	-1074 to 1582	8
1hz Treated pre vs post CNO	0.0018	-30678	-49322 to -12034	8
3hz Treated pre vs post CNO	0.0001	-33611	-48991 to -18231	8
6hz Treated pre vs post CNO	0.0002	-22653	-33592 to -11714	8
9hz Treated pre vs post CNO	0.0001	-11432	-16643 to -6221	8
12hz Treated pre vs post CNO	0.0004	-2562	-3891 to -1234	8
15hz Treated pre vs post CNO	0.0004	-2562	-3891 to -1234	8

Fig.4g

Two-way ANOVA with Sidak post hoc multiple comparison test
Comparing contrast sensitivity pre and post administration of CNO in treated and untreated eyes
95% CI

Comparison	P-value	Effect Size	95% CI	DF
0.128cycles/degree Untreated pre vs post CNO	0.903	0.0006222	-0.003137 to 0.004382	8
0.25 cycles per degree Untreated pre vs post CNO	0.894	0.0004667	-0.002223 to 0.003156	8
0.381 cycles per degree Untreated pre vs post CNO	0.9225	0.0001444	-0.0008383 to 0.001127	8
0.45 cycles per degree Untreated pre vs post CNO	0.0009	0.0008222	0.0003610 to 0.001283	8
0.128cycles/degree Treated pre vs post CNO	<0.0001	-0.008478	-0.01224 to -0.004718	8
0.25 cycles per degree Treated pre vs post CNO	0.0007	-0.004956	-0.007645 to -0.002266	8
0.381 cycles per degree Treated pre vs post CNO	0.0004	-0.001922	-0.002905 to -0.0009395	8
0.45 cycles per degree Treated pre vs post CNO	>0.9999	0	-0.0004612 to 0.0004612	8

Fig.5d

Two tailed paired t-test
Comparing visual acuity threshold pre and post retinal lesion induction
95% CI

Comparison	P-value	Effect Size	95% CI	DF

Acuity pre vs post lesion	<0.0001	-0.1204	-0.1616 to -0.07913	16

Fig.5i

One-way repeated measures ANOVA with Tukey post hoc multiple comparison test
Comparing acuity thresholds pre and post administration of CNO in treated and untreated eyes
95% CI

Comparison	P-value	Effect Size	95% CI	DF
Pre lesion vs post lesion Untreated	0.0013	0.1705	0.09949 to 0.2415	5
Post lesion vs post CNO Untreated	0.8526	-0.007667	-0.05329 to 0.03796	5
Pre lesion vs post CNO Untreated	0.0027	0.1628	0.08309 to 0.2426	5
Pre lesion vs post lesion Treated	0.0014	0.1547	0.08921 to 0.2201	5
Post lesion vs post CNO Treated	0.0053	-0.1358	-0.2131 to -0.05858	5
Pre lesion vs post CNO Treated	0.5924	0.01883	-0.04071 to 0.07838	5

Suppl.Fig.2

One-way ANOVA with Tukey post hoc multiple comparison test
Comparing equiluminant to low luminance spatial contrast sensitivity for each tested frequency
95% CI

Comparison	P-value	Effect Size	95% CI	DF
1.54 cycles/degree equiluminant vs low luminance	0.0057	0.2656	0.08259 to 0.4485	8
2.31 cycles/degree equiluminant vs low luminance	<0.0001	0.2678	0.1658 to 0.3698	8
3.09 cycles/degree equiluminant vs low luminance	0.0053	0.2167	0.06937 to 0.3640	8
6.17 cycles/degree equiluminant vs low luminance	0.0105	0.05667	0.01367 to 0.09966	8
9.26 cycles/degree equiluminant vs low luminance	0.3119	0.01	-0.005596 to 0.02560	8

Suppl.Fig.6

One-way ANOVA with Dunnet's post hoc multiple comparison test
Comparing the effect of an artifical scotoma on contrast sensitivity
95% CI

Comparison	P-value	Effect Size	95% CI	DF
equiluminant vs central scotoma	0.0002	0.5533	0.3948 to 0.7119	5
equiluminant vs peripheral scotoma	<0.0001	0.42	0.3304 to 0.5096	5

Suppl.Fig.8c

Two tailed paired t-test
Comparing contrast sensitivity proximal and distal to the retinal lesion
95% CI

Comparison	P-value	Effect Size	95% CI	DF
Proximal vs distal contrast sensitivity	0.0056	0.7638	0.3413 to 1.186	5

Suppl.Fig.12a

One-way repeated measures ANOVA withTukey post hoc multiple comparison test
Comparing contrast sensitivity in WT vs CNGA3-/- mice at different temporal frquencies
95% CI

Comparison	P-value	Effect Size	95% CI	DF
0.128 cycles/degree WT vs CNGA3-/-	<0.0001	-0.1472	-0.1785 to -0.1160	13
0.25 cycles/degree WT vs CNGA3-/-	<0.0001	-0.09135	-0.1226 to -0.06012	13
0.381 cycles/degree WT vs CNGA3-/-	0.8831	-0.0131	-0.04434 to 0.01813	13
0.45 cycles/degree WT vs CNGA3-/-	>0.9999	-0.002973	-0.03421 to 0.02826	13

Suppl.Fig.13

One-way ANOVA with Dunnet's post hoc multiple comparison test
Comparing the effect of different width and luminance annuli on contrast sensitivity

95% CI

Comparison	P-value	Effect Size	95% CI	DF
Equiluminant vs 0.05 degree low luminance annulus	0.9866	0.02222	-0.1329 to 0.1774	8
Equiluminant vs 0.1 degree low luminance annulus	0.0894	0.19	-0.02802 to 0.4080	8
Equiluminant vs 0.25 degree low luminance annulus	0.0028	0.2767	0.1138 to 0.4396	8
Equiluminant vs 0.5 degree low luminance annulus	<0.0001	0.4611	0.3271 to 0.5951	8
Equiluminant vs 1.5 degree low luminance annulus	<0.0001	0.54	0.4380 to 0.6420	8
Equiluminant vs 0.05 degree high luminance annulus	0.0013	0.2256	0.1072 to 0.3439	8
Equiluminant vs 0.1 degree high luminance annulus	<0.0001	0.3333	0.2162 to 0.4505	8
Equiluminant vs 0.25 degree high luminance annulus	<0.0001	0.4244	0.2756 to 0.5733	8
Equiluminant vs 0.5 degree high luminance annulus	<0.0001	0.5078	0.3594 to 0.6561	8
Equiluminant vs 1.5 degree high luminance annulus	<0.0001	0.5444	0.4010 to 0.6879	8
