# Supplementary material for: Lateral gain is impaired in macular degeneration and can be targeted to restore vision in mice
Source: Nat Commun. 2022 Apr 20;13:2159. doi: 10.1038/s41467-022-29666-x (PMC9021237; doi:10.1038/s41467-022-29666-x)
Supplement: Supplementary file 2 — Reporting Summary [file 41467_2022_29666_MOESM2_ESM.pdf]

nature research

Corresponding author(s): Robin Ali

Last updated by author(s): Jan 21, 2022

Reporting Summary

Nature Research wishes to improve the reproducibility of the work that we publish. This form provides structure for consistency and transparency in reporting. For further information on Nature Research policies, see Authors & Referees and the Editorial Policy Checklist .

Statistics

For all statistical analyses, confirm that the following items are present in the figure legend, table legend, main text, or Methods section.

n/a	Confirmed	
[ ]	[x]	The exact sample size (*n*) for each experimental group/condition, given as a discrete number and unit of measurement
[ ]	[x]	A statement on whether measurements were taken from distinct samples or whether the same sample was measured repeatedly
[ ]	[x]	The statistical test(s) used AND whether they are one- or two-sided<br>*Only common tests should be described solely by name; describe more complex techniques in the Methods section.*
[ ]	[x]	A description of all covariates tested
[ ]	[x]	A description of any assumptions or corrections, such as tests of normality and adjustment for multiple comparisons
[ ]	[x]	A full description of the statistical parameters including central tendency (e.g. means) or other basic estimates (e.g. regression coefficient) AND variation (e.g. standard deviation) or associated estimates of uncertainty (e.g. confidence intervals)
[ ]	[x]	For null hypothesis testing, the test statistic (e.g. *F*, *t*, *r*) with confidence intervals, effect sizes, degrees of freedom and *P* value noted<br>*Give P values as exact values whenever suitable.*
[x]	[ ]	For Bayesian analysis, information on the choice of priors and Markov chain Monte Carlo settings
[x]	[ ]	For hierarchical and complex designs, identification of the appropriate level for tests and full reporting of outcomes
[x]	[ ]	Estimates of effect sizes (e.g. Cohen's *d*, Pearson's *r*), indicating how they were calculated

Our web collection on statistics for biologists contains articles on many of the points above.

Software and code

Policy information about availability of computer code

Data collection	Matlab R2014b and R2018a (MathWorks inc); VisageMKII Stimulus Generator (Library version 8.201, VSG DLL version 1.271, Toolbox version 1.271, Cambridge Research Systems); Tobii Pro X120 (Tobii SDK 3.0, TobiPro); QUEST staircase algorithm (Watson and Pelli, 1983) Nidek MP-1 staircase algorithm and retinal tracking software (software version 1.7.8, Nidek); Spectralis OCT (Software version 6.9a, Heidelbergengineering).Cerebral Dynamics manufacurer's optomotor staircase algorithm (Cerebral Dynamics); ImageJ (ImageJ1.2a).
Data analysis	Matlab R2018a (MathWorks inc), Python 3.7 (Python Software Foundation). Toolboxes and custom scripts were used for MATLAB.

For manuscripts utilizing custom algorithms or software that are central to the research but not yet described in published literature, software must be made available to editors/reviewers. We strongly encourage code deposition in a community repository (e.g. GitHub). See the Nature Research guidelines for submitting code & software for further information.

Data

Policy information about availability of data

All manuscripts must include a data availability statement. This statement should provide the following information, where applicable:
- Accession codes, unique identifiers, or web links for publicly available datasets
- A list of figures that have associated raw data
- A description of any restrictions on data availability

Source data are provided with this paper. The data generated in this study is available in Supplementary information / Source Data file.

Field-specific reporting

Please select the one below that is the best fit for your research. If you are not sure, read the appropriate sections before making your selection.

Life sciences study design

All studies must disclose on these points even when the disclosure is negative.

Sample size	Sample size for patients was limited by the rarity of their mutations/conditions. As several of the experiments (e.g.: chemogenetic manipulation combined with optomotor tests) we based the selection of cohort size based on similar published work, making use of the same behavioral tests (Carvalho et al, 2011; Pearson et al, 2012; Nishiguchi et al., 2015);
Data exclusions	No collected data was excluded.
Replication	All data was obtained from at least two independent cohorts of subjects. All attempts at replication were successful and generated data.
Randomization	For experiments where multiple conditions were tested (temporal frequencies, luminance levels, spatial frequencies, surround luminance levels) presentation was pseudo-randomized, both for mouse and human subjects. In each experiment, all human studies participants underwent the same experimental protocol, testing multiple experimental conditions. Protocols did not require allocation in different experimental subgroups and therefore no allocation randomization was required. For mice, each mouse received injection of the vector in one eye and a sham injection in the other. The L/R eye injection in mice was pseudo-randomized.
Blinding	Mouse experiments were performed blind to the sham or treatment injection, performed by a different researcher. For human studies, data acquisition and data analysis were performed by two separate investigators and the latter was blind to the condition being analyzed. Researchers administering the test were not blind to the genotype of the subject being tested, because of the need to make provisions for the patient's visual needs. However, for each experiment all subjects underwent the same testing procedure. Testing was fully-automated, the assessment of visual thresholds was performed by the machine via a participant response box and a pre-set staircase procedure.

Reporting for specific materials, systems and methods

We require information from authors about some types of materials, experimental systems and methods used in many studies. Here, indicate whether each material, system or method listed is relevant to your study. If you are not sure if a list item applies to your research, read the appropriate section before selecting a response.

Materials & experimental systems

n/a	Involved in the study	
☐	☒	Antibodies
☒	☐	Eukaryotic cell lines
☒	☐	Palaeontology
☐	☒	Animals and other organisms
☐	☒	Human research participants
☒	☐	Clinical data

Methods

n/a	Involved in the study	
☒	☐	ChIP-seq
☒	☐	Flow cytometry
☒	☐	MRI-based neuroimaging

Antibodies

Antibodies used	Calbindin antibody, Brn3a antibody
Validation	The Calbindin antibody involved is widely used for staining of horizontal cells in the mouse retina (see for example Szikra, et al. 2014; Calbindin D-28k antibody (CB-38, Swant, dilution 1:500, validated for horizontal cells in Burger et al., 2020). anti-Brn3a antibody (Santa Cruz Biotechnology, dilution 1:500, sc-31984, C-20), previously validated for retinal ganglion cells by Nadal-Nicolas et al., 2009. See also Methods.

Animals and other organisms

Policy information about studies involving animals; ARRIVE guidelines recommended for reporting animal research

Laboratory animals	Mus Musculus. C57/B6 and Cnga3-/-, 5-15 week old, both Male and Female
Wild animals	No wild animals were used in the study.
Field-collected samples	No field collected animals were used in the study.
Ethics oversight	UCL, Inst. of Ophthalmology AWERB.

Note that full information on the approval of the study protocol must also be provided in the manuscript.

Human research participants

Policy information about studies involving human research participants

Population characteristics	Normal vision subjects included 12 males and 10 females between the ages of 18-52. Patients with specific conditions were aged 16-54.

Recruitment	Nomal vision subjects were recruited from within the Department. Patients with specific conditions were all patients registered at Moorfields Eye Hospital with a diagnosis of their condition based on multiple tests, including genotyping, ERG measurements and other functional tests. Psychophysics tests were based on 2-alternative forced choice tasks, specifically designed to remove the bias that the selection of specific participants might give to the experiment's results.
Ethics oversight	Moorfields Eye Hospital Ethics Committee (NHS REC reference: 11/H0703/10

Note that full information on the approval of the study protocol must also be provided in the manuscript.
