# Supplementary material for: Lateral gain is impaired in macular degeneration and can be targeted to restore vision in mice
Source: Nat Commun. 2022 Apr 20;13:2159. doi: 10.1038/s41467-022-29666-x (PMC9021237; doi:10.1038/s41467-022-29666-x)
Supplement: Supplementary file 3 — Peer Review File [file 41467_2022_29666_MOESM3_ESM.pdf]

nature portfolio

Peer Review File

Lateral gain is impaired in macular degeneration and can be targeted to restore vision in mouse

Open Access This file is licensed under a Creative Commons Attribution 4.0 International License, which permits use, sharing, adaptation, distribution and reproduction in any medium or format, as long as you give appropriate credit to the original author(s) and the source, provide a link to the Creative Commons license, and indicate if changes were made. In the cases where the authors are anonymous, such as is the case for the reports of anonymous peer reviewers, author attribution should be to 'Anonymous Referee' followed by a clear attribution to the source work. The images or other third party material in this file are included in the article's Creative Commons license, unless indicated otherwise in a credit line to the material. If material is not included in the article's Creative Commons license and your intended use is not permitted by statutory regulation or exceeds the permitted use, you will need to obtain permission directly from the copyright holder. To view a copy of this license, visit http://creativecommons.org/licenses/by/4.0/.

Editorial Note: This manuscript has been previously reviewed at another journal that is not operating a transparent peer review scheme. This document only contains reviewer comments and rebuttal letters for versions considered at *Nature Communications*.

Reviewers' Comments:

Reviewer #1:
Remarks to the Author:
1) In the Rizzi et al paper, the human psychophysics data demonstrated a long range adaptative mechanism. The paper attributes this adaptative mechanisms to H1 mediated lateral inhibition. However, this feedback was well characterized in the non-human primate retina by David Dacey (Dacey et al., 1996). He indicated that this feedback is "spatially restricted and cone type-specific adaptation over a broad illuminance range, in general agreement with the conclusions of psychophysical studies in human."(Lee et al., 1999). This spatially restricted adaptation is related to the morphology of H1 cells (100-300μm depending on eccentricity) and their low electrical coupling (Packer & Dacey, 2002). The morphology of H1 cells was similarly investigated in the human retina with very small dendritic arbors in the macula (Kolb et al., 1995).
2) Furthermore, if the adaptative mechanism due to the surround illumination was related to the horizontal cell axon as suggested in the figure 4a and 5a, then CSNB should not see any surround effect because their rod pathway is totally inoperant. However, Figure 2b clearly shows that CSNB patients have an adaptative mechanism.
3) The effect reported here in the transfected horizontal cells can be interpreted by an enhanced cone dynamic range. Hyperpolarizing horizonal cells is likely to enhance cone depolarization generating a higher dynamic for cone photoreceptor function and thus enhancing the ERG response. The expression of the construct is also present in the non-ablated retina as indicated in Fig. 5h. The relatively small level of expression is due to the counting at 1mm from the edge of the lesion but this represents a very significative expression all around the lesioned area, which is likely of less than 1mm diameter. As a consequence, the surrounding lesion area is likely more than double the surface of the lesioned area. The enhanced cone dynamic in this surrounding non-lesioned area could contribute therefore to the recovered spatial acuity.
4) The dopamine adaptative mechanism has a wide range surround effects due to the diffusion of the neurotransmitter and the large dendritic field of dopamine cells. Although the authors said they rephrased the paper on the corresponding subject, they still do not argue why their observation is not related to this well-known adaptative mechanism.

Dacey, D.M., Lee, B.B., Stafford, D.K., Pokorny, J. & Smith, V.C. (1996) Horizontal cells of the primate retina: cone specificity without spectral opponency. Science (New York, N.Y, 271, 656-659.

Kolb, H., Nelson, R.F., Ahnelt, P.K., Ortuno-Lizaran, I. & Cuenca, N. (1995) The Architecture of the Human Fovea. In Kolb, H., Fernandez, E., Nelson, R. (eds) Webvision: The Organization of the Retina and Visual System, Salt Lake City (UT).

Lee, B.B., Dacey, D.M., Smith, V.C. & Pokorny, J. (1999) Horizontal cells reveal cone type-specific adaptation in primate retina. Proceedings of the National Academy of Sciences of the United States of America, 96, 14611-14616.

Packer, O.S. & Dacey, D.M. (2002) Receptive field structure of H1 horizontal cells in macaque monkey retina. J Vis, 2, 272-292.

Reviewer #4:
Remarks to the Author:
In this manuscript Rizzi <i>et al.</i> find macular degeneration greatly reduces visual function, and lateral inhibition by horizontal cells may be a key aspect. The manuscript is elegant and the

study is solid.

Previous concerns:

<I>1. The authors interpret this finding to be due to lack of laterally projecting inhibitory circuits near the lesion (as opposed to the trivial explanation of greater disease near the lesion).</i>

The authors have improved the Figure and amended the text and their logic seems clear.

<i>2.In addition, the link between Stargardt's (the data collected) and AMD (the only mention of disease in the abstract) is tenuous at best. The choice of long-debunked citation #2 claiming a genetic link between the two diseases is not helpful.</i>

The authors have changed the citation and revised the manuscript text. The reasoning seems valid.

<i>3. Perception of a visual stimulus is well known to be strongly influenced by the luminance context. Here the authors use CSF in low and equiluminant environments to claim that the surround luminance through the horizontal cells acts as an adaptive mechanism of vision optimization. This claim needs to be first thoroughly evaluated by subspecialists in the field instead of being included in postage-stamp sized figures and extremely limited methodological details for a general medical audience.</i>

Manuscript has been modified to address the concerns. The logic is reasonable, and their experimental results agree with the gain/adaptation model .

<i>4. However, their data do not rule out involvement of different photoreceptor systems under low and equiluminant conditions in an eye with macular degeneration thus questioning the conjecture that it is only the lateral inhibition that is changing between the two conditions.</i>

This point has been clarified in the text and data added to address the concerns of the role of photoreceptors.

<i>5. They express the hM4Di receptor in a subset of horizontal cells with AAV2/8. After intraperitoneal CNO activation, they show minor improvements in electrophysiological and behavioral visual function driven by rod photoreceptors. It is not clear how these mouse results support the main thesis that cone-driven lateral inhibition can be used to improve cone vision.</i>

Experiments have been added to clarify the point, and the point resolved satisfactorily.

I have no further concerns with this manuscript.

Reviewer #5:
Remarks to the Author:
Lateral feedback is widespread in visual signal processing. Here, Rizzi et al provide evidence that loss of lateral signaling from horizontal cells to photoreceptors may contribute to visual deficits in geographic atrophies. The authors elegantly combine human psychophysics with work on mouse models with photoreceptor loss. Their work suggests that increasing lateral signalling from remaining horizontal cells may improve vision in mouse models for photoreceptor degeneration. Whether these findings can be extended to human patients remains to be seen.

Although I find the study interesting and highly relevant, I have several comments which need to be addressed.
1. The contribution of the authors seems occasionally overstated. What they call "lateral adaptive feedback" was earlier called "lateral gain control". For example, work from the Kamermans lab postulated (Kamermans et al., 1998; Kraaij et al., 1998) and later on proved that horizontal cells increase the gain of the photoreceptor synapse (VanLeeuwen et al., 2009). Similarly, already

Nelson et al. and Pflug et al. (1990) suggested a gain increase at the photoreceptor synapse induced by horizontal hyperpolarization. I suggest to insert this and the respective citations into the introduction and results part.
Related to this, as it is not strictly “lateral inhibition” but lateral “lateral gain control”, I suggest to change the wording.
2. CNGA3-/- mice: the authors state: “vision in these mice is mediated by rods and the absence of cone input deprives the rods of any H1 lateral feedback”. I doubt that this is true: First, Thoreson et al. (2008) showed that rods receive feedback from horizontal cell axon terminals. And second, Truempler et al. (2008) revealed that horizontal cell somata of CNGA3-/- mice show light responses which closely resemble rod responses. These responses reach the soma via rod-cone gap junctions. As signals can travel from the soma to the axon terminal via the axon, CNGA3-/- rods will receive lateral feedback from rods. The authors should discuss this and cite Truempler et al. (2008) when discussing cone-HC-rod pathways. I suggest to also alter the schematic in figure 4a accordingly.
3. The authors make use of psychophysical data from patients with specific photoreceptor mutations to identify the cell types involved in lateral adaptive feedback. However, I doubt that comparisons of contrast sensitivities across patients with different diseases are conclusive because retinal diseases cause various side effects and furthermore, lead to various degrees of remodeling which will affect results. These side effects vary from patient to patient. Whether the differences seen in BED patients vs. achromat patients (Fig. 3b) are really just due to differences in lateral signaling seems therefore highly questionable (in particular given the low n). I suggest to downtone the respective conclusions.
4. Title: the authors do not provide evidence for improving vision after macular degeneration. “following photoreceptor loss” is presumably more precise. See also my earlier comment above on “lateral inhibition”.

Reviewer 1's remarks to the authors:

1) In the Rizzi et al paper, the human psychophysics data demonstrated a long range adaptative mechanism. The paper attributes this adaptative mechanisms to H1 mediated lateral inhibition. However, this feedback was well characterized in the non-human primate retina by David Dacey (Dacey et al., 1996). He indicated that this feedback is "spatially restricted and cone type-specific adaptation over a broad illuminance range, in general agreement with the conclusions of psychophysical studies in human."(Lee et al., 1999). This spatially restricted adaptation is related to the morphology of H1 cells (100-300µm depending on eccentricity) and their low electrical coupling (Packer & Dacey, 2002). The morphology of H1 cells was similarly investigated in the human retina with very small dendritic arbors in the macula (Kolb et al., 1995).

We must respectfully point out that Reviewer 1 is misusing references to suggest that H1 cells only provide spatially restricted feedback. The experiments in Lee et al, 1999 are designed to study specifically local feedback from horizontal cells to cones and makes no attempt to study long range feedback to rods. Of course, dendritic mechanisms of feedback are restricted to the dendritic field and this does not lend weight to the argument. Similarly, the '96 paper by Dennis Dacey et al. aims to study the spectral selectivity of cone inputs to horizontal cells. Horizontal cell output onto rods is not studied in this paper. H1 cell feedback to rods has been studied and demonstrated in more recent literature (Trumpler et al., 2008; Szikra, et al., 2014; Joesch et al., 2016; Stroh et al., 2018) and our gain of function experiments in a rod-only $Cnga3^{-/-}$ mouse strongly support this.

2) Furthermore, if the adaptative mechanism due to the surround illumination was related to the horizontal cell axon as suggested in the figure 4a and 5a, then CSNB should not see any surround effect because their rod pathway is totally inoperant. However, Figure 2b clearly shows that CSNB patients have an adaptative mechanism.

In our manuscript we described in several instances how our data shows the impact of lateral feedback onto both cone and rod photoreceptors, not just rods. For example, (lines 131-135, old MS; lines 134-138 new MS): "In patients with cone-only vision (congenital stationary night blindness, CSNB), we found that engaging lateral feedback with an equiluminant surround improved contrast sensitivity, demonstrating the importance of lateral feedback in optimizing cone-mediated vision." We also discussed this in relation to our observations in patients with Bornholm eye disease, (lines 137-139, old MS; lines 140-142 new MS): "In these subjects, we made the interesting observation that engagement of lateral input improved not only cone-mediated vision, but also rod-mediated vision." We return to this point in the discussion, (lines 253-255, old MS; lines 257-259, new MS): "By combining silent substitution methods[24] with naturally occurring functional 'knock-out' conditions, we found that lateral feedback optimizes both cone-mediated and rod-mediated vision." For our gain of function experiments in $Cnga3^{-/-}$ mice (which lack cone function), we were able to study the effect on rod-mediated vision. These results provide a powerful demonstration that H1 feedback does improve rod vision. However, this by no means excludes future studies on other types of lateral feedback.

3) The effect reported here in the transfected horizontal cells can be interpreted by an enhanced cone dynamic range. Hyperpolarizing horizonal cells is likely to enhance cone depolarization generating a higher dynamic for cone photoreceptor function and thus enhancing the ERG response. The expression of the construct is also present in the non-ablated retina as indicated in Fig. 5h. The relatively small level of expression is due to the

counting at 1mm from the edge of the lesion but this represents a very significative expression all around the lesioned area, which is likely of less than 1mm diameter. As a consequence, the surrounding lesion area is likely more than double the surface of the lesioned area. The enhanced cone dynamic in this surrounding non-lesioned area could contribute therefore to the recovered spatial acuity.

Horizontal cell mediated enhancement of cone function, for example via the axon of H2 horizontal cells, may provide an additional improvement in vision in our experiments. This would fit nicely with our observations made in patient studies (see above answer to point 2) and would greatly strengthen the potential benefit of our therapeutic strategy in patients with macular degeneration. However, we do want to stress that our experiments in $Cnga3^{-/-}$ mice demonstrate that H1 feedback onto rods does improve vision. This cannot be dismissed, and it is therefore very likely that improvement in rod photoreceptor function contributes to the observed improvement in vision. As we have data to directly support this argument, we believe that this remains the most conservative logic and appropriate interpretation for our findings.

Local horizontal dendrite-cone pedicle interactions outside of lesion area are unaffected by the lesion. Hence the cone dynamic range is naturally optimized by the intact circuitry. Further hyperpolarisation of the horizontal cell in this case would not be able to enhance the cone dynamic range further and therefore this cannot offer an explanation for the improved vision we observed.

4) The dopamine adaptative mechanism has a wide range surround effects due to the diffusion of the neurotransmitter and the large dendritic field of dopamine cells. Although the authors said they rephrased the paper on the corresponding subject, they still do not argue why their observation is not related to this well-known adaptative mechanism.

At no point in the manuscript do we aim to exclude particular cell types or neurotransmitters from contributing to our observed adaptive effects. Rather, we aim to identify one cell that, among several, could contribute to this effect, and that could be targeted to mediate an improvement in vision. This is perhaps best summarized in the discussion (lines 270-272, old MS; lines 276-278 new MS): “Although several classes of laterally-projecting cells may be involved in the effect we observe[2,3], several features make H1 horizontal cells a good candidate for gain-of-function experiments.”

Reviewer 4’s remarks to the authors:

In this manuscript Rizzi *et al.* find macular degeneration greatly reduces visual function, and lateral inhibition by horizontal cells may be a key aspect. The manuscript is elegant and the study is solid.

Previous concerns:

1. The authors interpret this finding to be due to lack of laterally projecting inhibitory circuits near the lesion (as opposed to the trivial explanation of greater disease near the lesion).

The authors have improved the Figure and amended the text and their logic seems clear.

2.In addition, the link between Stargardt’s (the data collected) and AMD (the only mention of

disease in the abstract) is tenuous at best. The choice of long-debunked citation #2 claiming a genetic link between the two diseases is not helpful.

The authors have changed the citation and revised the manuscript text. The reasoning seems valid.

3. Perception of a visual stimulus is well known to be strongly influenced by the luminance context. Here the authors use CSF in low and equiluminant environments to claim that the surround luminance through the horizontal cells acts as an adaptive mechanism of vision optimization. This claim needs to be first thoroughly evaluated by subspecialists in the field instead of being included in postage-stamp sized figures and extremely limited methodological details for a general medical audience.

Manuscript has been modified to address the concerns. The logic is reasonable, and their experimental results agree with the gain/adaptation model.

4. However, their data do not rule out involvement of different photoreceptor systems under low and equiluminant conditions in an eye with macular degeneration thus questioning the conjecture that it is only the lateral inhibition that is changing between the two conditions.

This point has been clarified in the text and data added to address the concerns of the role of photoreceptors.

5. They express the hM4Di receptor in a subset of horizontal cells with AAV2/8. After intraperitoneal CNO activation, they show minor improvements in electrophysiological and behavioral visual function driven by rod photoreceptors. It is not clear how these mouse results support the main thesis that cone-driven lateral inhibition can be used to improve cone vision.

Experiments have been added to clarify the point, and the point resolved satisfactorily.

I have no further concerns with this manuscript.

We thank Reviewer 4 for the positive assessment of our amendments.

Reviewer #5 (Remarks to the Author):

Lateral feedback is widespread in visual signal processing. Here, Rizzi et al provide evidence that loss of lateral signaling from horizontal cells to photoreceptors may contribute to visual deficits in geographic atrophies. The authors elegantly combine human psychophysics with work on mouse models with photoreceptor loss. Their work suggests that increasing lateral signalling from remaining horizontal cells may improve vision in mouse models for photoreceptor degeneration. Whether these findings can be extended to human patients remains to be seen.

Although I find the study interesting and highly relevant, I have several comments which need to be addressed.

1. The contribution of the authors seems occasionally overstated. What they call “lateral adaptive feedback” was earlier called “lateral gain control”. For example, work from the Kamermans lab postulated (Kamermans et al., 1998; Kraaij et al., 1998) and later on proved that horizontal cells increase the gain of the photoreceptor synapse (VanLeeuwen et al.,

2009). Similarly, already Nelson et al. and Pflug et al. (1990) suggested a gain increase at the photoreceptor synapse induced by horizontal hyperpolarization. I suggest to insert this and the respective citations into the introduction and results part.
Related to this, as it is not strictly “lateral inhibition” but lateral “lateral gain control”, I suggest to change the wording.

We have taken onboard this suggestion by Reviewer #5. We are happy to stick to the original terminology with ‘lateral gain control’ and have amended the wording throughout the text. We have also inserted the suggested citations.

2. CNGA3-/- mice: the authors state: “vision in these mice is mediated by rods and the absence of cone input deprives the rods of any H1 lateral feedback”. I doubt that this is true: First, Thoreson et al. (2008) showed that rods receive feedback from horizontal cell axon terminals. And second, Truempler et al. (2008) revealed that horizontal cell somata of CNGA3-/- mice show light responses which closely resemble rod responses. These responses reach the soma via rod-cone gap junctions. As signals can travel from the soma to the axon terminal via the axon, CNGA3-/- rods will receive lateral feedback from rods. The authors should discuss this and cite Truempler et al. (2008) when discussing cone-HC-rod pathways. I suggest to also alter the schematic in figure 4a accordingly.

To be more accurate in our description of the Cnga3-/- model we have changed the wording from “deprives rods of any H1 lateral feedback”, to “deprives rods of cone-driven H1 lateral feedback”. We think it is a fair comment to say that H1 cells could receive rod input to their dendrites via rod-cone gap junctions. However, this input would arise from a rod that has different light response properties to a cone and that also lacks cone-driven gain control. As a result, this circuitry will not replicate the cone-driven gain control of normal physiology. As suggested by the Reviewer, we now discuss the findings by Truempler et al. (2008) in lines 290-294 (new manuscript): “In Cnga3$^{-/-}$ mice, rods can provide input to H1 cells via their gap junction connections with neighboring cones[34]. However, this input differs substantially from a cone-driven input and will be unable to give appropriate gain control at mesopic/photopic light levels.”. We have also modified Fig.4a to show this connection.

3. The authors make use of psychophysical data from patients with specific photoreceptor mutations to identify the cell types involved in lateral adaptive feedback. However, I doubt that comparisons of contrast sensitivities across patients with different diseases are conclusive because retinal diseases cause various side effects and furthermore, lead to various degrees of remodeling which will affect results. These side effects vary from patient to patient. Whether the differences seen in BED patients vs. achromat patients (Fig. 3b) are really just due to differences in lateral signaling seems therefore highly questionable (in particular given the low n). I suggest to downtone the respective conclusions.

We agree with the reviewer that, in isolation, small numbers for each patient condition need to be taken with care. It is however reassuring that data from all patients together provided a coherent picture. We have now amended the text in several places, to tone down the assertiveness of our conclusions related to this data, as suggested by Reviewer 5.

4. Title: the authors do not provide evidence for improving vision after macular degeneration. “following photoreceptor loss” is presumably more precise. See also my earlier comment above on “lateral inhibition”.

We have amended the title to be in line with the suggestions by Reviewer 5.

Reviewers' Comments:

Reviewer #5:
Remarks to the Author:
My concerns have been fully addressed. I congratulate the authors to this elegant study.
